# Association between plasma phospholipid saturated fatty acids and metabolic markers of lipid, hepatic, inflammation and glycaemic pathways in eight European countries: a cross-sectional analysis in the EPIC-InterAct study

**DOI:** 10.1186/s12916-017-0968-4

**Published:** 2017-11-17

**Authors:** Ju-Sheng Zheng, Stephen J. Sharp, Fumiaki Imamura, Albert Koulman, Matthias B. Schulze, Zheng Ye, Jules Griffin, Marcela Guevara, José María Huerta, Janine Kröger, Ivonne Sluijs, Antonio Agudo, Aurelio Barricarte, Heiner Boeing, Sandra Colorado-Yohar, Courtney Dow, Miren Dorronsoro, Pia T. Dinesen, Guy Fagherazzi, Paul W. Franks, Edith J. M. Feskens, Tilman Kühn, Verena Andrea Katzke, Timothy J. Key, Kay-Tee Khaw, Maria Santucci de Magistris, Francesca Romana Mancini, Elena Molina-Portillo, Peter M. Nilsson, Anja Olsen, Kim Overvad, Domenico Palli, Jose Ramón Quirós, Olov Rolandsson, Fulvio Ricceri, Annemieke M. W. Spijkerman, Nadia Slimani, Giovanna Tagliabue, Anne Tjonneland, Rosario Tumino, Yvonne T. van der Schouw, Claudia Langenberg, Elio Riboli, Nita G. Forouhi, Nicholas J. Wareham

**Affiliations:** 1 MRC Epidemiology Unit, University of Cambridge School of Clinical Medicine, Box 285, Institute of Metabolic Science, Cambridge Biomedical Campus, Cambridge, CB2 0QQ UK; 2 MRC Elsie Widdowson Laboratory, Cambridge, UK; 3 NIHR BRC Nutritional Biomarker Laboratory, Cambridge, UK; 4 Department of Molecular Epidemiology, German Institute of Human Nutrition Potsdam-Rehbruecke, Nuthetal, Germany; 5 Department of Biochemistry, University of Cambridge, Cambridge, UK; 6 Navarra Public Health Institute (ISPN), Pamplona, Spain; 7 Navarra Institute for Health Research (ldiSNA), Pamplona, Spain; 8 CIBER Epidemiology and Public Health (CIBERESP), Madrid, Spain; 9 Department of Epidemiology, Murcia Regional Health Council, IMIB-Arrixaca, Murcia, Spain; 10 University Medical Center Utrecht, Utrecht, The Netherlands; 11 Unit of Nutrition and Cancer, Cancer Epidemiology Research Program, Catalan Institute of Oncology-IDIBELL, L’Hospitalet de Llobregat, Barcelona, Spain; 12 Department of Epidemiology, German Institute of Human Nutrition Potsdam-Rehbruecke, Nuthetal, Germany; 13 Research Group on Demography and Health, National Faculty of Public Health, University of Antioquia, Medellín, Colombia; 14 INSERM U1018, Center for Research in Epidemiology and Population Health, Villejuif, France; 15 University Paris-Saclay, University Paris-Sud, Villejuif, France; 16 Gustave Roussy, F-94805 Villejuif, France; 17 Public Health Division of Gipuzkoa, San Sebastian, Spain; 18 Department of Cardiology, Aalborg University Hospital, Aalborg, Denmark; 19 Lund University, Malmö, Sweden; 20 Umeå University, Umeå, Sweden; 21 Wageningen University, Wageningen, Netherlands; 22 German Cancer Research Center (DKFZ), Division of Cancer Epidemiology, Heidelberg, Germany; 23 Cancer Epidemiology Unit, Nuffield Department of Population Health, University of Oxford, Oxford, UK; 24 Department of Public Health and Primary Care, University of Cambridge, Cambridge, UK; 25 A.O.U. Federico II, Naples, Italy; 26 Escuela Andaluza de Salud Pública, Instituto de Investigación Biosanitaria ibs.GRANADA, Hospitales Universitarios de Granada/Universidad de Granada, Granada, Spain; 27 Danish Cancer Society Research Center, Copenhagen, Denmark; 28 Department of Public Health, Section for Epidemiology, Aarhus University, Aarhus, Denmark; 29 Cancer Research and Prevention Institute (ISPO), Florence, Italy; 30 Public Health Directorate, Asturias, Spain; 31 Department of Clinical and Biological Sciences, University of Turin, Turin, Italy; 32 Unit of Epidemiology, Regional Health Service ASL TO3, Grugliasco, Turin, Italy; 33 National Institute for Public Health and the Environment (RIVM), Bilthoven, The Netherlands; 34 International Agency for Research on Cancer, Lyon, France; 35 Fondazione IRCCS Istituto Nazionale dei Tumori, Milan, Italy; 36 Cancer Registry and Histopathology Department, “Civic M.P. Arezzo” Hospital, ASP, Ragusa, Italy; 37 School of Public Health, Imperial College London, London, UK

**Keywords:** Saturated fatty acids, Odd-chain, Even-chain, Very-long-chain, Metabolic markers, Lipids, Hepatic, Glycaemic, Inflammation

## Abstract

**Background:**

Accumulating evidence suggests that individual circulating saturated fatty acids (SFAs) are heterogeneous in their associations with cardio-metabolic diseases, but evidence about associations of SFAs with metabolic markers of different pathogenic pathways is limited. We aimed to examine the associations between plasma phospholipid SFAs and the metabolic markers of lipid, hepatic, glycaemic and inflammation pathways.

**Methods:**

We measured nine individual plasma phospholipid SFAs and derived three SFA groups (odd-chain: C15:0 + C17:0, even-chain: C14:0 + C16:0 + C18:0, and very-long-chain: C20:0 + C22:0 + C23:0 + C24:0) in individuals from the subcohort of the European Prospective Investigation into Cancer and Nutrition (EPIC)-InterAct case-cohort study across eight European countries. Using linear regression in 15,919 subcohort members, adjusted for potential confounders and corrected for multiple testing, we examined cross-sectional associations of SFAs with 13 metabolic markers. Multiplicative interactions of the three SFA groups with pre-specified factors, including body mass index (BMI) and alcohol consumption, were tested.

**Results:**

Higher levels of odd-chain SFA group were associated with lower levels of major lipids (total cholesterol (TC), triglycerides, apolipoprotein A-1 (ApoA1), apolipoprotein B (ApoB)) and hepatic markers (alanine transaminase (ALT), aspartate transaminase (AST), gamma-glutamyl transferase (GGT)). Higher even-chain SFA group levels were associated with higher levels of low-density lipoprotein cholesterol (LDL-C), TC/high-density lipoprotein cholesterol (HDL-C) ratio, triglycerides, ApoB, ApoB/A1 ratio, ALT, AST, GGT and CRP, and lower levels of HDL-C and ApoA1. Very-long-chain SFA group levels showed inverse associations with triglycerides, ApoA1 and GGT, and positive associations with TC, LDL-C, TC/HDL-C, ApoB and ApoB/A1. Associations were generally stronger at higher levels of BMI or alcohol consumption.

**Conclusions:**

Subtypes of SFAs are associated in a differential way with metabolic markers of lipid metabolism, liver function and chronic inflammation, suggesting that odd-chain SFAs are associated with lower metabolic risk and even-chain SFAs with adverse metabolic risk, whereas mixed findings were obtained for very-long-chain SFAs. The clinical and biochemical implications of these findings may vary by adiposity and alcohol intake.

**Electronic supplementary material:**

The online version of this article (doi:10.1186/s12916-017-0968-4) contains supplementary material, which is available to authorized users.

## Background

There has been ongoing interest in the association between circulating saturated fatty acids (SFAs) and cardio-metabolic diseases such as cardiovascular diseases and type 2 diabetes. Recent evidence has highlighted that different individual circulating SFAs have discordant associations with cardio-metabolic diseases [1–7]. For example, we and others reported that circulating odd-chain SFAs (C15:0 (pentadecanoic acid) and C17:0 (heptadecanoic acid)) and very-long-chain SFAs (C20:0 (arachidic acid), C22:0 (behenic acid), C23:0 (tricosanoic acid) and C24:0 (lignoceric acid)) were inversely associated with incident type 2 diabetes, while circulating even-chain SFAs (C14:0 (myristic acid), C16:0 (palmitic acid) and C18:0 (stearic acid)) were positively associated [1–4]. In a meta-analysis, circulating C17:0 was inversely associated with coronary heart disease, while no association was found for any of C14:0, C15:0, C16:0 or C18:0 [5]. Other studies showed that higher circulating C16:0 was associated with higher risk of atrial fibrillation and heart failure, whereas other SFAs (C18:0, C20:0, C22:0 and C24:0) showed inverse or non-significant associations with these cardiac outcomes [6, 7].

The pathogenesis of cardio-metabolic diseases involves various metabolic pathways and intermediate metabolic markers, including markers of lipid metabolism, liver function, chronic inflammation and glycaemic homeostasis [8]. Clarifying the associations of individual circulating SFAs with these metabolic markers in different pathways will help in the understanding of the roles of individual SFAs in disease aetiology. Previous studies of circulating SFAs with intermediate metabolic markers were inconsistent or not comprehensive and analysed using different lipid fractions (phospholipid fraction, erythrocyte membrane, etc.) [9–17]. For instance, odd-chain SFAs C15:0 and C17:0 from erythrocyte membranes were positively associated with low-density lipoprotein cholesterol (LDL-C) and C17:0 was inversely associated with high-density lipoprotein cholesterol (HDL-C) in men, while C15:0 was positively associated with HDL-C in women in a European study [11]. Both circulating odd-chain and even-chain SFAs showed heterogeneous associations with glycaemic markers, which were studied in different lipid fractions [3, 14, 15, 17–19]. Circulating very-long-chain SFAs (C20:0, C22:0 and C24:0) had mixed associations with different metabolic markers, including a positive association with LDL-C, an inverse association with triglycerides (TG) and an inverse association (C24:0 only) with C-reactive protein (CRP) and insulin resistance [2]. The inconsistency in the existing literature may reflect the heterogeneity of sample sizes, ranging from 38 [20] to 3004 participants [3]. Furthermore, fatty acids were measured using different methods in different lipid fractions across different studies, which might contribute to the inconsistent results in the existent literature. This adds to the rationale for measuring fatty acids in one single lipid fraction (such as circulating phospholipids) in a large multicentre study with diverse population characteristics to characterise robust associations of fatty acids with metabolic markers.

Therefore, evaluating fatty acid profiles in populations in eight European countries, we aimed to examine the associations of individual circulating plasma phospholipid SFAs and corresponding SFA groups with several metabolic markers of lipid metabolism, liver function, chronic inflammation and glycaemic control.

## Methods

### Study design and population

These analyses used data from the subcohort of the European Prospective Investigation into Cancer and Nutrition (EPIC)-InterAct study. EPIC-InterAct is a case-cohort study of type 2 diabetes nested within eight countries of the EPIC cohort study, namely France, Italy, Spain, UK, the Netherlands, Germany, Sweden and Denmark [21]. Briefly, from the entire cohort of 340,234 participants with blood samples (baseline years, 1991–1998) in the eight countries, we ascertained incident diabetes cases and selected a random subcohort of 16,835 participants. Participants with prevalent diabetes at baseline were excluded (n = 681) [21]. A further 235 participants with no available fatty acid data were also excluded. Thus, a total of 15,919 participants were included in this study. All participants provided written informed consent and the study was approved by the local ethics committee in the participating centres and the Internal Review Board of the International Agency for Research on Cancer.

### Laboratory measurements

Plasma phospholipid fatty acids were measured between 2010 and 2012 at the Medical Research Council Human Nutrition Research (Cambridge, UK) from non-fasting plasma samples stored at baseline (1993–1998) at –196 °C (–150 °C in Denmark). We considered plasma phospholipid fatty acid compositions as stable because stability has been confirmed at –80 °C or below over long-term storage [22]. The laboratory staff were blind to any information about the participants. The assay method and quality control of the fatty acid measurement have been described elsewhere [23]. A total of 37 fatty acids were identified by comparing their retention time with those of commercial standards. All the fatty acids were expressed as percentage of total phospholipid fatty acid (mol%). Nine SFAs had a relative concentration higher than 0.05%. Data quality was assessed using quality control samples across all of the batches, which showed that the SFAs and their coefficients of variation were C14:0 (9.4%), C15:0 (11.9%), C16:0 (1.6%), C17:0 (4.2%), C18:0 (2.0%), C20:0 (15.3%), C22:0 (10.3%), C23:0 (18.9%) and C24:0 (14.7%).

Serum metabolic markers were measured at Stichting Ingenhousz Laboratory (Etten-Leur, Netherlands), from samples stored at –196 °C (–80 °C in Umeå). These metabolic markers included lipid markers (total cholesterol (TC), HDL-C TG, apolipoprotein A-1 (ApoA1) and apolipoprotein B (ApoB)), liver function markers (alanine transaminase (ALT), aspartate transaminase (AST), gamma glutamyl transferase (GGT)), and an inflammatory marker (C-reactive protein (CRP)). All assays were performed using a Cobas® (Roche Diagnostics, Mannheim, Germany) assay on a Roche Hitachi Modular P analyser. Several other lipid markers were calculated based on the measured lipids, namely LDL-C based on the Friedewald formula [24], TC/HDL-C (a ratio of TC to HDL-C) and ApoB/A1 (a ratio of ApoB to ApoA1). A marker of glycaemic control, haemoglobin A1c (HbA1c), was measured at Stichting Ingenhousz Laboratory in the erythrocyte fraction from samples stored at –196 °C (–80 °C in Umeå) using the Tosoh-G8 analyser (Tosoh Bioscience, Japan). The assay detection ranges for the metabolic marker measurements were TC (0.08–20.7 mmol/L), HDL-C (0.08–3.1 mmol/L), TG (0.05–11.3 mmol/L), ApoA1 (7.14–143 μmol/L), ApoB (0.39–7.8 μmol/L), ALT (4–600 U/L), AST (4–800 U/L), GGT (3–1200 U/L), HbA1c (20–140 mmol/mol) and CRP (0.1–20 mg/L). Quality control was based on the Westgard rules [25]. All the metabolic markers were winsorised based on the 0.1 and 99.9th percentiles to minimise influence of potential outliers.

### Diet and lifestyle measurements

The assessment of habitual diet during the past 12 months was undertaken using self- or interviewer-administered, country-specific validated dietary questionnaires [26]. Based on the standardised EPIC Nutrient Database [27], total energy and nutrient intake were calculated. At baseline, we used standardised health and lifestyle questionnaires to assess demographics, smoking status, medical history and educational level. Physical activity was assessed using a brief questionnaire at baseline, which divided participants into four physical activity groups, namely inactive, moderately inactive, moderately active or active [21].

### Statistical analysis

We used Stata version 14 for all analyses. We analysed nine individual SFAs (C14:0, C15:0, C16:0, C17:0, C18:0, C20:0, C22:0, C23:0, C24:0) and three SFA groups – odd-chain SFA (sum of 15:0 and 17:0), even-chain SFA (sum of 14:0, 16:0 and 18:0) and very-long-chain SFA (sum of C20:0, C22:0, C23:0, C24:0). The groupings of the SFAs were based on those used in a previous analysis of EPIC-InterAct [1]. Spearman correlation coefficients between the individual SFAs were calculated. As dependent variables, we evaluated eight lipid markers (TC, HDL-C, TC/HDL-C, LDL-C, TG, ApoB, ApoA1, ApoB/A1), three liver function markers (ALT, AST and GGT), one glycaemic marker (HbA1c) and one inflammatory marker (CRP). Variables with skewed distribution were log-transformed before analysis (TC/HDL-C, TG, ALT, AST, GGT and CRP). In the primary analysis (based on complete case analysis), we used linear regression to estimate country-specific associations per 1 standard deviation (SD, calculated in the overall subcohort) of each SFA with each of the 13 metabolic markers, and combined the estimated associations across countries using random effects meta-analysis. Associations were expressed as the difference in metabolic marker (in SD units) per 1 SD difference in each fatty acid. A *P* value threshold for significance of 0.00068 was defined based on the number of tests performed, accounting for correlations among exposures and outcomes, calculated using the method of Li and Ji [28]. Confidence intervals (CI) were also estimated, which were consistent with this *P* value threshold.

Using linear regression, we adjusted for the potential confounders as follows: Model 1 – age, sex and centre; Model 2 – as Model 1 plus body mass index (BMI); Model 3 – as Model 2 plus physical activity (inactive, moderately inactive, moderately active or active), smoking status (never, former or current), alcohol drinking (never, 0 to < 6, 6 to < 12, 12 to < 24, and ≥ 24 g/day), educational level (none, primary school, technical or professional school, secondary school, or higher education) and total energy intake (kcal/day). Model 3 was extended to test the potential interaction of the three SFA groups (even-chain, odd-chain and very-long-chain SFAs) with age, sex, BMI, alcohol drinking and physical activity. For these pre-specified interaction tests on the multiplicative scale to investigate potential biological variation, a *P* value threshold of 0.0004 was used, calculated using the same method as above [28].

We performed sensitivity analyses based on Model 3, where (3a) included a variety of dietary factors as covariates (dietary intakes of carbohydrates, dairy, red and processed meat, fruits and vegetables, olive oil and vegetable oil); (3b) excluded participants with HbA1c ≥ 6.5%; (3c) included self-reported heart disease, stroke and cancer as covariates; (3d) excluded participants with self-reported heart disease, stroke or cancer; (3e) excluded people with hyperlipidaemia (self-reported hyperlipidaemia or treatment for hyperlipidaemia); (3f) included hyperlipidaemia status as a covariate; (3g) was a mutual adjustment for the other SFA groups; (3h) excluded participants with very heavy alcohol drinking (>95th percentile of the present population (>50.2 g/d)); (3i) excluded participants with CRP ≥ 95.2 nmol/L (equivalent to 10 mg/L); and (3j) replaced BMI with waist circumference as a marker of adiposity.

## Results

### Population characteristics

The mean age of the InterAct subcohort participants was 52.3 years (SD 9.2 years, range 20–77 years). Participants who were men, older or with higher BMI had relatively higher concentrations of even-chain SFAs. Women or those with lower BMI had higher odd-chain and very-long-chain SFA levels (Table 1). In addition, women had a more favourable metabolic profile than men, such as higher levels of HDL-C and lower levels of TC/HDL-C, TG and liver function markers. Older participants and those with a higher BMI had higher levels of TC, LDL-C, TC/HDL-C, TG, ApoB, ApoB/A1, ALT, AST, GGT, HbA1c and CRP. The distributions of these metabolic markers varied across different participating countries and across different SFA groups (Additional file 1: Table S1, Table S2). The odd-chain SFA group was negatively correlated with the even-chain SFA group, while being positively correlated with a very-long-chain SFA group (Additional file 1: Table S3). The correlations of the three SFA groups with various food sources in InterAct have been reported previously [1].

**Table 1:** Distribution of plasma metabolic biomarkers and saturated fatty acid groups in the subcohort (*n* = 15919) and by sex, age and BMI: EPIC-InterAct study

	n	Overall Subcohort	Sex		Age (years)			BMI (kg/m^2^)		
			Men (*n* = 6002)	Women (*n* = 9917)	<40 (*n* = 1524)	40–60 (*n* = 10919)	≥60 (*n* = 3476)	<25 (*n* = 7052)	25–30 (*n* = 6255)	≥30 (*n* = 2502)
TC (mmol/L)	15304	5.93 (1.11)	5.92 (1.08)	5.94 (1.13)	5.25 (0.97)	5.92 (1.07)	6.29 (1.13)	5.8 (1.08)	6.05 (1.12)	6.04 (1.13)
HDL-C (mmol/L)	15306	1.50 (0.42)	1.30 (0.36)	1.61 (0.41)	1.48 (0.39)	1.50 (0.42)	1.50 (0.43)	1.63 (0.43)	1.41 (0.38)	1.33 (0.36)
LDL-C (mmol/L)	15125	3.83 (1.00)	3.90 (0.96)	3.79 (1.02)	3.29 (0.88)	3.82 (0.98)	4.11 (1.02)	3.66 (0.98)	3.97 (1.00)	3.96 (0.97)
TC/HDL-C^a^	15304	4.00 (3.21–5.00)	4.63 (3.77–5.70)	3.67 (3.00–4.53)	3.50 (2.87–4.42)	4.00 (3.21–5.00)	4.27 (3.47–5.33)	3.53 (2.91–4.4)	4.31 (3.50–5.40)	4.57 (3.73–5.64)
TG (mmol/L)^a^	15303	1.1 (0.8–1.6)	1.3 (0.9–1.9)	1.0 (0.7–1.4)	0.9 (0.6–1.2)	1.1 (0.8–1.6)	1.3 (0.9–1.8)	0.9 (0.7–1.3)	1.2 (0.9–1.8)	1.4 (0.9–2)
ApoA1 (μmol/L)	15308	54.8 (10.4)	50.2 (8.9)	57.6 (10.2)	53.2 (10.5)	54.8 (10.3)	55.6 (10.4)	56.9 (10.7)	53.5 (9.9)	52.5 (9.5)
ApoB (μmol/L)	15295	1.94 (0.5)	2.03 (0.49)	1.89 (0.5)	1.66 (0.44)	1.94 (0.49)	2.10 (0.5)	1.83 (0.48)	2.02 (0.5)	2.06 (0.49)
ApoB/A1	15295	0.04 (0.01)	0.04 (0.01)	0.03 (0.01)	0.03 (0.01)	0.04 (0.01)	0.04 (0.01)	0.03 (0.01)	0.04 (0.01)	0.04 (0.01)
ALT (μkat/L)^a^	15274	0.32 (0.23–0.42)	0.38 (0.3–0.52)	0.28 (0.22–0.37)	0.27 (0.2–0.38)	0.32 (0.23–0.43)	0.32 (0.25–0.4)	0.27 (0.22–0.35)	0.33 (0.25–0.45)	0.38 (0.28–0.53)
AST (μkat/L)^a^	15068	0.45 (0.38–0.52)	0.48 (0.42–0.57)	0.43 (0.37–0.50)	0.40 (0.35–0.47)	0.45 (0.38–0.52)	0.47 (0.42–0.55)	0.43 (0.37–0.5)	0.45 (0.40–0.53)	0.47 (0.40–0.57)
GGT (μkat/L)^a^	15295	0.33 (0.23–0.53)	0.47 (0.33–0.73)	0.27 (0.22–0.40)	0.27 (0.20–0.37)	0.33 (0.23–0.53)	0.37 (0.27–0.55)	0.28 (0.22–0.42)	0.37 (0.25–0.6)	0.43 (0.28–0.68)
HbA1c (mmol/mol)	15711	36.2 (5.0)	36.4 (5.3)	36.1 (4.8)	33.6 (3.7)	36.0 (5.0)	38.0 (4.8)	35.5 (4.2)	36.4 (4.9)	37.9 (6.6)
CRP (nmol/L)^a^	15298	10.3 (5.05–22.1)	10.2 (5.24–20.9)	10.4 (4.95–22.8)	7.71 (3.43–18.4)	9.81 (4.86–20.9)	13.4 (6.67–27.2)	6.86 (3.52–14.4)	11.7 (6.29–22.9)	21.1 (10.9–39.6)
Odd-chain SFAs (mol%)	15919	0.63 (0.13)	0.58 (0.13)	0.65 (0.13)	0.63 (0.11)	0.62 (0.14)	0.63 (0.14)	0.65 (0.13)	0.61 (0.13)	0.60 (0.13)
Even-chain SFAs (mol%)	15919	44.6 (1.22)	44.8 (1.12)	44.5 (1.27)	44.2 (1.09)	44.6 (1.21)	44.8 (1.26)	44.4 (1.22)	44.7 (1.20)	44.9 (1.20)
Very-long-chain SFAs (mol%)	15919	0.70 (0.20)	0.69 (0.20)	0.71 (0.20)	0.70 (0.19)	0.70 (0.18)	0.72 (0.24)	0.71 (0.19)	0.70 (0.22)	0.69 (0.16)

^a^These metabolic markers were presented as median (interquartile range); all other metabolic markers or SFA groups were presented as mean (SD)

*TC* total cholesterol, *HDL-C* high-density lipoprotein cholesterol, *LDL-C* low-density lipoprotein cholesterol, *TG* triglycerides, *ApoA1* apolipoprotein A-1, *ApoB* apolipoprotein B, *ALT* alanine transaminase, *AST* aspartate transaminase, *GGT* gamma glutamyl transferase, *HbA1c* haemoglobin A1c, *CRP* C-reactive protein, *SFA* saturated fatty acid

### Association of plasma phospholipid SFAs with metabolic markers

#### Odd-chain SFAs

**Fig. 1:**
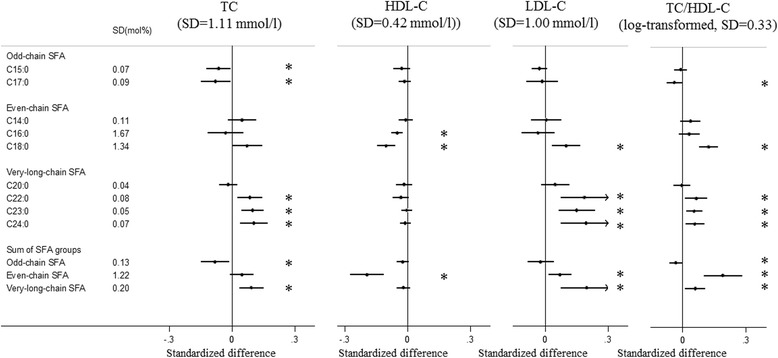
Associations of plasma phospholipid saturated fatty acids (SFAs) with total and types of cholesterol: EPIC-InterAct study. Standardised difference is the difference (in SD units of the metabolic marker) per 1 SD of each SFA. The error bars represent confidence intervals, corrected for multiple testing based on a familywise error rate of 5%. * Indicates that the *P* value for the association was < 0.00068. Estimates were based on random effects meta-analysis of country-specific estimates from a linear regression adjusted for age, sex, centre, BMI, physical activity, smoking status, alcohol drinking, educational level and total energy intake. TC/HDL-C was log-transformed. *TC* total cholesterol, *HDL-C* high-density lipoprotein cholesterol, *LDL-C* low-density lipoprotein cholesterol

**Fig. 2:**
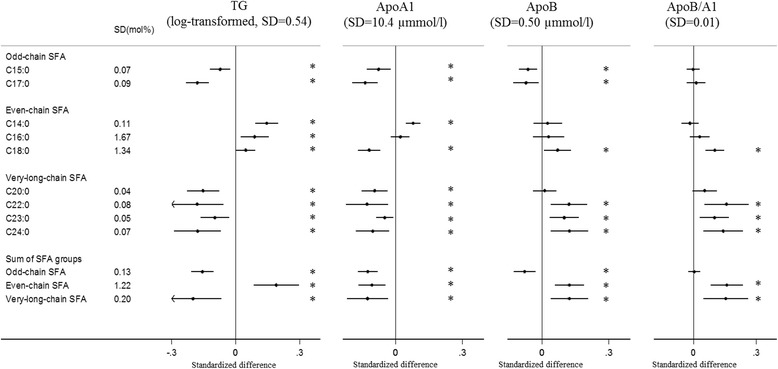
Associations of plasma phospholipid saturated fatty acids (SFAs) with triglycerides and apolipoproteins: EPIC-InterAct study. Standardised difference is the difference (in SD unit of the metabolic marker) per 1 SD of each SFA. The error bars represent confidence intervals, corrected for multiple testing based on a family-wise error rate of 5%. * Indicates that the *P* value for the association was < 0.00068. Estimates were based on random effects meta-analysis of country-specific estimates from a linear regression adjusted for age, sex, centre, BMI, physical activity, smoking status, alcohol drinking, educational level and total energy intake. *ApoA1* apolipoprotein A-1, *ApoB* apolipoprotein B

**Fig. 3:**
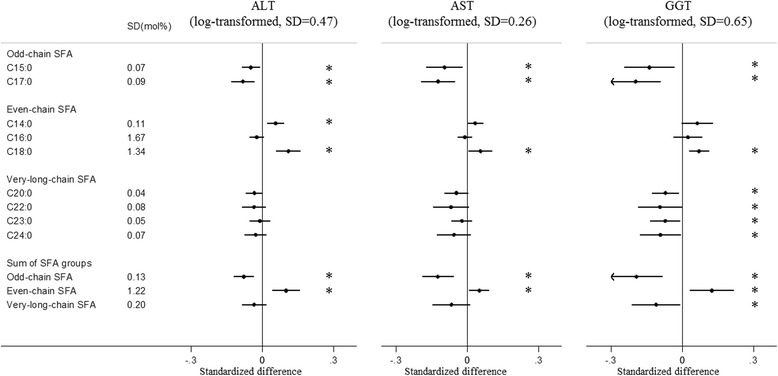
Associations of plasma phospholipid saturated fatty acids (SFAs) with circulating liver enzymes: EPIC-InterAct study. Standardised difference is the difference (in SD unit of the metabolic marker) per 1 SD of each SFA. The error bars represent confidence intervals, corrected for multiple testing based on a family-wise error rate of 5%. * Indicates that the *P* value for the association was < 0.00068. Estimates were based on random effects meta-analysis of country-specific estimates from a linear regression adjusted for age, sex, centre, BMI, physical activity, smoking status, alcohol drinking, educational level and total energy intake. All the ALT, AST and GGT were log-transformed. *ALT* alanine transaminase, *AST* aspartate transaminase, *GGT* gamma glutamyl transferase

**Fig. 4:**
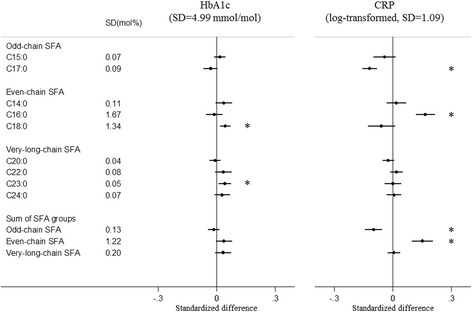
Associations of plasma phospholipid saturated fatty acids (SFAs) with haemoglobin A1c and C-reactive protein: EPIC-InterAct study. Standardised difference is the difference (in SD unit of the metabolic marker) per 1 SD of each SFA. The error bars represent confidence intervals, corrected for multiple testing based on a family-wise error rate of 5%. * Indicates that the *P* value for the association was < 0.00068. Estimates were based on random effects meta-analysis of country-specific estimates from a linear regression adjusted for age, sex, centre, BMI, physical activity, smoking status, alcohol drinking, educational level and total energy intake. CRP was log-transformed. *TG* triglycerides, *HbA1c* haemoglobin A1c

**Fig. 5:**
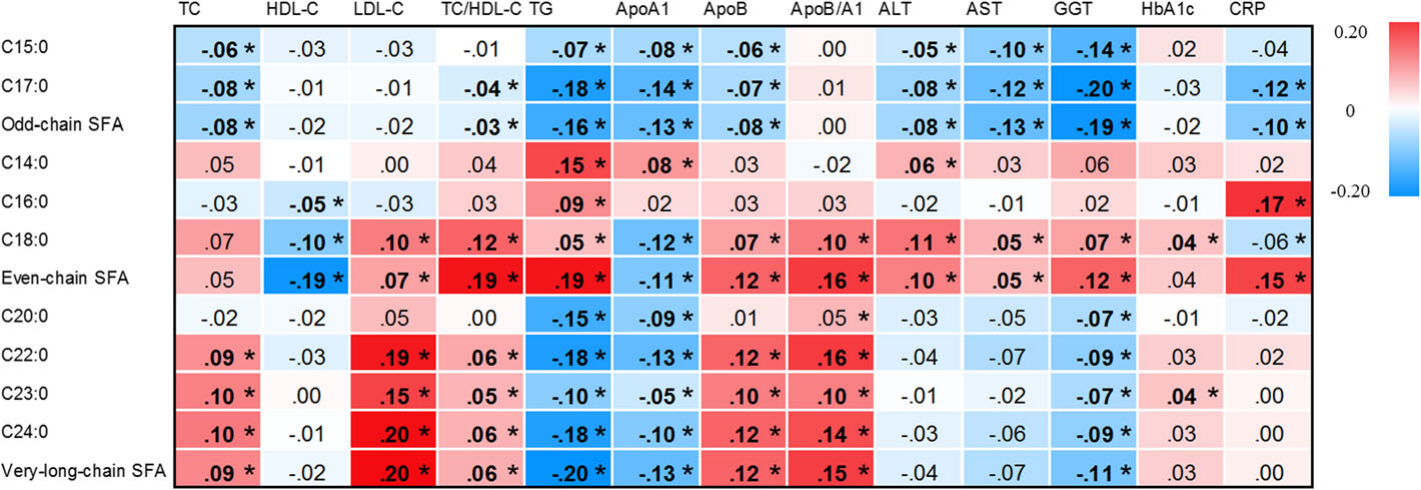
Summary of the associations of plasma phospholipid saturated fatty acids (SFAs) with metabolic markers: EPIC-InterAct study. Value in each cell represented standardised difference (in SD unit) of metabolic markers per 1 SD difference in each variable of plasma phospholipid SFAs. Values in bold (with *) indicate that the *P* value for the association was < 0.00068. All the values are expressed in red scale for different levels of positive associations and blue scale for different levels of negative associations

Both C15:0 and C17:0 were inversely associated with several lipid (TC, TG, ApoA1, ApoB) and liver function markers (ALT, AST and GGT). In addition, C17:0 was inversely associated with TC/HDL-C and CRP (Figs. 1, 2, 3, 4 and 5, Additional file 1: Table S4). The odd-chain SFA group (C15:0 + C17:0) was inversely associated with most metabolic markers; there was no evidence of an association with HDL-C, LDL-C, ApoB/A1 or HbA1c (Fig. 5).

#### Even-chain SFAs

Circulating C14:0 was positively associated with circulating levels of TG, ApoA1 and ALT, but there was no association with the other metabolic markers. Circulating C16:0 was inversely associated with HDL-C, and positively associated with TG and CRP. C18:0 was inversely associated with HDL-C and ApoA1, and positively associated with LDL-C, TC/HDL-C, TG, ApoB, ApoB/A1, ALT, AST, GGT and HbA1c. The even-chain SFA group was significantly positively associated with 9 of the 13 metabolic markers (Figs. 1, 2, 3, 4 and 5, Additional file 1: Table S5).

#### Very-long-chain SFAs

Circulating C20:0 was inversely associated with TG, ApoA1 and GGT; while C22:0, C23:0 and C24:0 showed consistently positive associations with TC, LDL-C, TC/HDL-C, ApoB and ApoB/A1, and inverse associations with TG, ApoA1 and GGT (Figs. 1, 2, 3, 4 and 5, Additional file 1: Table S6). C23:0 was also positively associated with HbA1c (Fig. 4). As a group, very-long-chain SFAs had the strongest inverse association with TG, while other associations were similar to those of C24:0 (Fig. 5).

#### Sensitivity and interaction analyses

Most of the sensitivity analyses did not substantially affect the results (Additional file 1: Table S7). There was heterogeneity between countries in some of the SFAs and metabolic marker associations, such as LDL-C, TG and GGT, although the directions of the associations were generally consistent across countries (Additional file 1: Figure S1).

Results of interaction analyses showed that, in non-alcohol drinkers, there was no evidence of an association of the odd-chain SFA group with TC, ApoA1, ALT or AST, while in the higher alcohol consumption group, there was an inverse association of the odd-chain SFA group with TC, ApoA1, ALT, AST and GGT (Additional file 1: Figure S2). Similarly, a stronger inverse association of the very-long-chain SFA group with HDL-C and ApoA1 was found among high alcohol drinkers, with no significant association among non-drinkers. There was a suggestion that the association of each SFA group with each metabolic marker was stronger in those with higher BMI. In addition, the association of odd-chain SFAs with some metabolic markers (TC, AST, ALT and GGT) appeared to be stronger in men than in women.

## Discussion

In a large cohort of European adults, we identified distinct patterns of association between different SFAs and metabolic markers. Briefly, odd-chain SFAs had favourable associations, while even-chain SFAs had adverse associations with most metabolic markers; there were mixed patterns of association with very-long-chain SFAs. We found that both odd-chain SFAs (C15:0 and C17:0) had consistent inverse associations with lipid markers (TC, TG, ApoA1 and ApoB) and liver function markers (ALT, AST and GGT). The odd-chain SFA C17:0 was additionally inversely associated with another lipid marker (TC/HDL-C ratio) and with the chronic inflammatory marker (CRP). In contrast, there were positive associations between the even-chain SFA group (sum of C14:0, C16:0 and C18:0) and several lipids (LDL-C, TC/HDL-C, TG, ApoB, ApoB/A1), liver function markers (ALT, AST, GGT) and CRP, and inverse associations with HDL-C and ApoA1. These associations were mainly driven by C18:0, except for CRP (only associated with C16:0). C14:0 and C16:0 showed heterogeneous associations with different markers. For very-long-chain SFAs (C20:0, C22:0, C23:0 and C24:0), there were heterogeneous findings – the group was inversely associated with two lipid markers (TG, ApoA1) and one liver function marker (GGT), but positively associated with other lipid markers (TC, LDL-C, TC/HDL-C, ApoB and ApoB/A1).

Eight of the 13 metabolic markers included in the analysis were lipid markers. Higher LDL-C and TG and lower HDL-C are known risk factors for cardiovascular diseases [29–31]. ApoA1 is abundant in anti-atherogenic HDL particles, while ApoB is part of the atherogenic non-HDL particles (e.g., LDL). Both ApoB/A1 and TC/HDL-C represent a balance of the anti-atherogenic and atherogenic lipoprotein particles, and are considered better predictors of the future cardiovascular disease risk than the individual lipids [32, 33]. Previous literature has reported heterogeneous findings on the association of SFAs with these lipid markers [2, 3, 11–14, 17]. We did not find an association between odd-chain SFAs and the ratios of ApoB/A1 or TC/HDL-C (after adjustment for the other SFA groups), suggesting that odd-chain SFAs might not be independently associated with anti-atherogenic properties. Instead, they may reflect the associations of the other SFA groups, as both even-chain and very-long-chain SFAs were positively associated with ApoB/A1 and TC/HDL-C in the present study and the three groups were correlated. There was a strong inverse association between very-long-chain SFAs and TG, which was consistent with the inverse association between very-long-chain SFAs and type 2 diabetes and cardiovascular diseases [1, 7]. The findings that very-long-chain SFAs had positive associations with atherogenic lipid markers (e.g. LDL-C, TC/HDL-C and ApoB/A1) were consistent with previous studies on LDL-C levels [2, 34]. In addition, very-long-chain SFAs in erythrocytes were inversely associated with LDL particle size [34], and smaller LDL particles are considered more atherogenic than larger LDL particles [35].

Several studies [12, 36, 37] examined the associations of SFAs with liver function markers, with mixed findings. Even-chain SFAs were positively associated with hepatic markers in one study [12], but not associated in several other studies [36, 37], with no evidence that odd-chain or very-long-chain SFAs were associated with these markers. In our study, odd-chain SFAs were inversely associated, C18:0 was positively associated with the three hepatic markers included, and very-long-chain SFAs were inversely associated with GGT. These findings were consistent with the direction of the association found for cardio-metabolic disease endpoints, including type 2 diabetes and cardiovascular diseases [1–4, 7, 13, 38].

The associations of SFAs with HbA1c and CRP were modest. Only C18:0 and C23:0 had weak positive associations with HbA1c. The positive association between C23:0 and HbA1c was contradictory to the inverse association of C23:0 with incident type 2 diabetes previously found in this population [1]. However, the relative concentration of C23:0 was lowest among the nine SFAs, and the current cross-sectional association with HbA1c may not be comparable to results from association with subsequent incident disease. One odd-chain SFA (C17:0) and one even-chain SFA (C16:0) were, respectively, inversely and positively associated with CRP, an inflammatory marker and risk factor of cardio-metabolic diseases [39]. The findings for CRP were consistent with several prior US studies in which none of C14:0, C15:0 or C18:0 were associated with CRP, while C16:0 was positively associated [3, 13]. Of note, previous studies only reported associations for a maximum of three of the individual SFAs with CRP [3, 13], while the present study provided a more comprehensive analysis across nine individual SFAs.

No studies have previously reported the interaction between alcohol or BMI and SFAs on metabolic outcomes. We observed consistent patterns of interaction for alcohol intake and BMI with SFA groups for the metabolic markers, with stronger SFAs and metabolic marker associations in groups with higher reported alcohol consumption or higher BMI. The mechanism behind these interactions is not clear. It may be that higher alcohol intake (which stimulates de novo lipogenesis) [40] or higher BMI is associated with higher absolute circulating SFA concentrations. Therefore, associations per SD SFA relative concentration might be larger among higher alcohol intake or BMI groups (with higher absolute SFA levels). Nevertheless, these interactions suggested that dietary exposure and adiposity should be taken into account in evaluating the relationship between individual SFAs and metabolic risk factors.

Results of the present study may have important biological implications linking SFAs and metabolic diseases. First, we found that odd-chain SFAs were favourably associated with markers of lipids (with exceptions for HDL-C and ApoA1), liver function and chronic inflammation, which we postulated might contribute to the inverse association of odd-chain SFA with cardio-metabolic diseases [1, 5]. It was recently shown that C15:0 and C17:0 may have different origins [41] and that C15:0 is a marker of exogenous origin dairy fat intake, while C17:0 is substantially biosynthesised endogenously. Another study suggested that both C15:0 and C17:0 are also biomarkers of dietary fibre intake [42]. These results suggest that the observed associations in this study indirectly reflect the impact of dietary dairy product and fibre intake on cardio-metabolic disease. Second, total even-chain SFA levels were positively associated with cardio-metabolic diseases potentially through all the tested pathways in this study, while individual even-chain SFAs appeared to have differential roles in these pathways. For example, only C16:0 was associated with the chronic inflammatory marker, CRP, in agreement with several in vitro studies [43–45]. The observed associations of even-chain SFAs with metabolic markers might partly reflect the effect of deregulation of de novo lipogenesis [46], as all of the three even-chain SFAs are within the de novo lipogenesis pathway, and these fatty acids were positively associated with risk of metabolic disorders [3, 12]. Third, very-long-chain SFAs were favourably associated with markers of lipids and liver function, which may be part of the mechanisms behind the inverse association of this group of SFAs with cardio-metabolic diseases, such as type 2 diabetes [1]. These observations are plausible as previous animal and cell culture studies [47] have suggested that ceramides containing very-long-chain SFAs show favourable effects on biological pathways (e.g. hepatocyte physiology and pathophysiology), compared with ceramides containing C16:0. In contrast, very-long-chain SFAs were positively associated with atherogenic lipid markers. Therefore, a TG or TG-related pathway may be a more reliable indicator linking plasma phospholipid very-long-chain SFAs and cardio-metabolic diseases compared to other lipid markers.

The strengths of our study include its large sample size of nearly 16,000 participants from eight European countries with different dietary/lifestyle backgrounds, and objective measurement of nine individual plasma phospholipid SFAs. The main limitation of this study is that the associations are cross-sectional, and therefore temporality of the associations is unclear and reverse causation cannot be ruled out. Additionally, as is common with all observational research, despite our attempts to adjust for a comprehensive range of potential confounders, residual confounding cannot be excluded. Future longitudinal studies relating changes in SFAs to change in metabolic markers as well as to the metabolome will be informative, and use of genetic Mendelian randomisation analysis and pharmacological or dietary interventions altering fatty acid profiles will help with causal inference. Another limitation is the possibility of false positive results among the large number of analyses performed, although we used a conservative approach to correct for multiple testing to minimise this possibility. Furthermore, potential measurement errors may have occurred because of the long-term storage of our plasma samples and may have biased our findings toward the null. Results from the phospholipid fatty acids may not be able to represent those from other lipid fractions, such as cholesterol and TG, and potential heterogeneity may exist across different lipid fractions. Finally, only one marker of inflammation (CRP) was available in our study, and future studies on this topic would benefit from an expanded panel of inflammatory cytokines, acute phase reactants and adipokines to further advance our understanding.

## Conclusions

Our analyses suggest that odd-chain SFAs are inversely associated with several metabolic markers in pathways of lipid metabolism, liver function and chronic inflammation, thereby potentially contributing to their inverse associations with cardio-metabolic diseases. Further, even-chain SFAs are unfavourably associated with metabolic markers in all the tested pathways, including lipid metabolism, liver function, glycaemic control and chronic inflammation, with inconsistent associations for different individual even-chain SFAs. Very-long-chain SFAs are inversely associated with TG and markers of liver function, which potentially contributes to their inverse associations with cardio-metabolic diseases, while their positive associations with atherogenic lipid markers warrants further study. Of note, associations of the three SFA groups with metabolic markers may vary by dietary alcohol intake and adiposity. The cross-sectional findings of the present study warrant further investigations in a prospective study. Taken together, these findings help to further our understanding for the role of SFAs in metabolic pathways and disease aetiology.

## Additional file

Additional file 1: Table S1. Population characteristics by the eight participating countries: EPIC-InterAct study. **Table S2.** Distribution of plasma metabolic markers by quartiles of plasma phospholipid saturated fatty acid (SFA) groups: EPIC-InterAct study. **Table S3.** Pairwise correlation of plasma phospholipid SFAs in the subcohort of EPIC-InterAct study. **Table S4.** Association of plasma phospholipid odd-chain SFAs (per 1 SD difference) with metabolic markers: EPIC-InterAct study. **Table S5.** Association of plasma phospholipid even-chain SFAs (per 1 SD difference) with metabolic markers: EPIC-InterAct study. **Table S6.** Association of plasma phospholipid very-long-chain SFAs (per 1 SD difference) with metabolic markers: EPIC-InterAct study. **Table S7.** Sensitivity analysis for the association of plasma phospholipid SFA groups and metabolic markers: EPIC-InterAct study. **Figure S1.** Association of plasma phospholipid SFA groups with LDL-C, TG and GGT by countries: EPIC-InterAct study. **Figure S2.** Association of plasma phospholipid SFA groups with metabolic markers by subgroups of alcohol intake, BMI and sex: EPIC-InterAct study. (DOCX 321 kb)

## Abbreviations

ALT: alanine transaminase
ApoA1: apolipoprotein A-1
ApoB: apolipoprotein B
AST: aspartate transaminase
CRP: C-reactive protein
EPIC: European Prospective Investigation into Cancer and Nutrition
GGT: gamma glutamyl transferase
HbA1c: haemoglobin A1c
HDL-C: high-density lipoprotein cholesterol
LDL-C: low-density lipoprotein cholesterol
SFA: saturated fatty acid
TC: total cholesterol
TG: triglycerides
