# Supplementary material for: Association between plasma phospholipid saturated fatty acids and metabolic markers of lipid, hepatic, inflammation and glycaemic pathways in eight European countries: a cross-sectional analysis in the EPIC-InterAct study
Source: BMC Med. 2017 Nov 17;15:203. doi: 10.1186/s12916-017-0968-4 (PMC5691386; doi:10.1186/s12916-017-0968-4)

**Title: Association between plasma phospholipid saturated fatty acids and metabolic markers of lipid, hepatic, inflammation and glycaemic pathways in eight European countries: a cross-sectional analysis in the EPIC-InterAct Study**

**Table S1 Population characteristics by the eight participating countries: EPIC-InterAct Study**

**Table S2 Distribution of plasma metabolic markers by quartiles of plasma phospholipid saturated fatty acid (SFA) groups: EPIC-InterAct Study**

**Table S3 Pairwise correlation of plasma phospholipid saturated fatty acids in the subcohort of EPIC-InterAct study**

**Table S4 Association of plasma phospholipid odd-chain saturated fatty acids (per 1 SD difference) with metabolic markers: EPIC-InterAct Study**

**Table S5 Association of plasma phospholipid even-chain saturated fatty acids (per 1 SD difference) with metabolic markers: EPIC-InterAct Study**

*

**Table S6 Association of plasma phospholipid very-long-chain saturated fatty acids (per 1 SD difference) with metabolic markers: EPIC-InterAct Study**

**Table S7 Sensitivity analysis for the association of plasma phospholipid saturated fatty acid groups and metabolic markers: EPIC-InterAct Study**

**Figure S1 Association of plasma phospholipid saturated fatty acid groups with LDL-C, TG and GGT by countries: EPIC-InterAct Study.** Standardised difference is the difference (in SD unit of the metabolic marker) per 1 SD of the saturated fatty acid (SFA) group: odd-chain SFA (C15:0 + C17:0), even-chain SFA (C14:0 + C16:0 + C18:0), very-long-chain SFA (C20:0 + C22:0 + C23:0 + C24:0). TG and GGT were log-transformed. Forest plot for the associations of SFA groups with LDL-C, TG and GGT were presented in this figure as high heterogeneity across countries were observed for the three metabolic markers.

**Figure S2 Association of plasma phospholipid saturated fatty acid groups with metabolic markers by subgroups of alcohol intake, BMI and sex: EPIC-InterAct Study.** Standardised difference is the difference (in SD unit of the metabolic marker) per 1 SD of the saturated fatty acid (SFA) group: odd-chain SFA (C15:0 + C17:0), even-chain SFA (C14:0 + C16:0 + C18:0), very-long-chain SFA (C20:0 + C22:0 + C23:0 + C24:0). Based on the significant interaction observed (p<0.0004 for multiple testing), we presented standardized difference of the metabolic marker per 1 SD of fatty acid by alcohol intake, BMI and sex subgroups. TG, TC/ HDL-CALT, AST, GGT and CRP were log-transformed. TC, total cholesterol; HDL-C, high-density lipoprotein cholesterol; LDL-C, low-density lipoprotein cholesterol; TG, triglycerides; ApoA1, apolipoprotein A-1; ApoB, apolipoprotein B; ALT, alanine transaminase; AST, aspartate transaminase; GGT, gamma glutamyl transferase; HbA1c, haemoglobin A1c; CRP, C-reactive protein.

**Table S1 Population characteristics by the eight participating countries: EPIC-InterAct Study**

|  | France (n=587) | Italy  (n=2006) | Spain (n=3555) | UK  (n=1310) | Netherlands (n=1500) | Germany (n=2045) | Sweden (n=2826) | Denmark (n=2090) |
| --- | --- | --- | --- | --- | --- | --- | --- | --- |
| Age (y) | 56.3 (6.5) | 50.1 (7.9) | 49.1 (7.8) | 56.9 (10.8) | 52 (11) | 50.3 (8.5) | 53.7 (10.5) | 56.6 (4.4) |
| BMI (kg/m^2^) | 23.1 (3.7) | 25.8 (4.1) | 28.2 (4.2) | 25.4 (3.8) | 25.3 (3.9) | 25.8 (4.1) | 25.0 (3.9) | 26.0 (3.9) |
| Waist circumference (cm) | 75.9 (9) | 83.8 (11.7) | 91.8 (11.8) | 83.4 (12.2) | 82.9 (11.2) | 85.9 (12.8) | 84 (12.9) | 89.2 (12.6) |
| Sex |  |  |  |  |  |  |  |  |
| Men | 0 | 33.3 | 38.1 | 38.5 | 18.7 | 41.6 | 43.3 | 53.5 |
| Women | 100 | 66.7 | 61.9 | 61.5 | 81.3 | 58.4 | 56.7 | 46.5 |
| Alcohol consumption |  |  |  |  |  |  |  |  |
| 0 g/d | 14.7 | 16.6 | 34.6 | 14.0 | 16.8 | 3.8 | 13.6 | 2.1 |
| >0-<6 g/d | 36.6 | 33 | 22.8 | 43.6 | 38.0 | 37.2 | 45.5 | 22.1 |
| 6-<12 g/d | 15.8 | 10.8 | 9.8 | 19.7 | 14.5 | 18.3 | 19.6 | 20.2 |
| 12-<24 g/d | 20.3 | 15.6 | 12.0 | 14.0 | 16.2 | 19.3 | 14.9 | 21.7 |
| ≥24 g/d | 12.6 | 22.4 | 20.8 | 8.0 | 14.1 | 21.5 | 6.4 | 33.7 |
| Physical activity |  |  |  |  |  |  |  |  |
| Inactive | 17.9 | 29 | 37.4 | 29.2 | 7.7 | 16.2 | 24.2 | 10.4 |
| Moderately inactive | 38.2 | 37.2 | 33.4 | 30.8 | 25.7 | 36.7 | 33.4 | 29.8 |
| Moderately active | 34.6 | 17.1 | 17.7 | 20.0 | 22.7 | 26.8 | 25.2 | 24.3 |
| Active | 9.4 | 15.0 | 11.5 | 14.0 | 37.7 | 20.3 | 17.0 | 35.4 |
| Educational level |  |  |  |  |  |  |  |  |
| None | 0.5 | 1.1 | 32.2 | 0 | 0 | 0.7 | 0.4 | 0 |
| Primary | 10.6 | 46.0 | 40.9 | 27.2 | 18.1 | 20 | 36.1 | 32.6 |
| Technical or professional | 0 | 12.2 | 7.8 | 29.9 | 30.4 | 37.7 | 25.1 | 36.9 |
| Secondary | 46.5 | 24.8 | 7.1 | 9.3 | 29.3 | 6.7 | 16.8 | 9.9 |
| Higher education | 38.2 | 14.0 | 11.2 | 18.9 | 21.5 | 35.0 | 21.3 | 20.4 |
| Smoking status |  |  |  |  |  |  |  |  |
| Never | 64.7 | 44 | 55.1 | 47.9 | 39.8 | 47.1 | 45 | 33.5 |
| Former | 21.3 | 27.3 | 17.3 | 32.9 | 32.2 | 32.5 | 27.4 | 30.4 |
| Current | 8.9 | 26.6 | 27.6 | 13.3 | 27.6 | 20.3 | 26.8 | 36 |
| TC (mmol/l) | 5.98 (0.92) | 5.9 (1.1) | 5.8 (1.06) | 6.05 (1.16) | 6.15 (1.18) | 5.9 (1.11) | 5.98 (1.16) | 5.94 (1.07) |
| HDL-C (mmol/l) | 1.85 (0.41) | 1.48 (0.4) | 1.48 (0.38) | 1.47 (0.43) | 1.49 (0.41) | 1.51 (0.43) | 1.45 (0.41) | 1.5 (0.46) |
| LDL-C (mmol/l) | 3.71 (0.85) | 3.90 (1) | 3.77 (0.95) | 3.89 (1.04) | 4.01 (1.05) | 3.73 (1.02) | 3.9 (1.04) | 3.73 (0.98) |
| TC/ HDL-C ^§^ | 3.21 (2.74-3.86) | 4 (3.29-4.94) | 3.93 (3.2-4.83) | 4.14 (3.25-5.36) | 4.18 (3.36-5.23) | 3.94 (3.14-5) | 4.2 (3.33-5.27) | 4 (3.19-5.18) |
| TG (mmol/l) ^§^ | 0.8 (0.6-1.1) | 1.0 (0.7-1.4) | 1.0 (0.7-1.4) | 1.3 (0.9-1.9) | 1.2 (0.9-1.7) | 1.2 (0.8-1.7) | 1.2 (0.9-1.7) | 1.3 (0.9-1.9) |
| ApoA1 (µmol/l) | 62.1 (9.77) | 55.4 (10.5) | 54.5 (9.06) | 55.1 (10.3) | 56 (10.36) | 57.1 (10.7) | 52 (10.0) | 53.1 (10.9) |
| ApoB (µmol/l) | 1.84 (0.4) | 1.94 (0.48) | 1.92 (0.48) | 1.97 (0.52) | 2.01 (0.52) | 1.89 (0.5) | 2.02 (0.52) | 1.91 (0.48) |
| ApoB/A1 | 0.03 (0.01) | 0.04 (0.01) | 0.04 (0.01) | 0.04 (0.01) | 0.04 (0.01) | 0.03 (0.01) | 0.04 (0.01) | 0.04 (0.01) |
| ALT (µkat/l) ^§^ | 0.27 (0.22-0.33) | 0.32 (0.23-0.42) | 0.32 (0.23-0.42) | 0.32 (0.25-0.42) | 0.30 (0.23-0.40) | 0.32 (0.23-0.45) | 0.32 (0.23-0.43) | 0.3 (0.23-0.42) |
| AST (µkat/l) ^§^ | 0.45 (0.4-0.52) | 0.43 (0.37-0.5) | 0.45 (0.38-0.52) | 0.50 (0.43-0.57) | 0.43 (0.38-0.52) | 0.42 (0.37-0.52) | 0.45 (0.38-0.52) | 0.45 (0.4-0.53) |
| GGT (µkat/l) ^§^ | 0.28 (0.22-0.38) | 0.32 (0.22-0.48) | 0.3 (0.22-0.47) | 0.32 (0.23-0.5) | 0.32 (0.23-0.47) | 0.35 (0.25-0.6) | 0.35 (0.25-0.57) | 0.43 (0.28-0.72) |
| HbA1c (mmol/mol) | 36.2 (3.67) | 36.5 (5.48) | 35.6 (4.99) | 35.8 (4.14) | 36.1 (5.43) | 35.2 (5.15) | 37.8 (4.74) | 36.3 (4.61) |
| CRP (nmol/l) ^§^ | 6.86 (3.62-16) | 10.1 (4.86-21.1) | 11.1 (5.71-23.2) | 9.9 (4.67-21.4) | 11.7 (5.33-25.7) | 9.62 (4.86-19.7) | 9.62 (4.67-21.4) | 10.9 (5.43-23.9) |
| Odd-chain SFAs (mol%) | 0.78 (0.16) | 0.62 (0.12) | 0.62 (0.14) | 0.67 (0.14) | 0.66 (0.14) | 0.61 (0.13) | 0.61 (0.11) | 0.59 (0.12) |
| Even-chain SFAs (mol%) | 45.4 (1.75) | 44.3 (1.09) | 44.2 (1.13) | 44.7 (1.5) | 44.6 (1.42) | 44.5 (0.89) | 44.9 (1.13) | 44.9 (1.0) |
| Very-long-chain SFAs (mol%) | 0.77 (0.15) | 0.68 (0.18) | 0.69 (0.14) | 0.79 (0.24) | 0.85 (0.35) | 0.63 (0.17) | 0.7 (0.15) | 0.65 (0.13) |

^§^ These metabolic markers were presented as median (interquartile range), other variables were presented as mean (SD) or percentage;

TC, total cholesterol; HDL-C, high-density lipoprotein cholesterol; LDL-C, low-density lipoprotein cholesterol; TG, triglycerides; ApoA1, apolipoprotein A-1; ApoB, apolipoprotein B; ALT, alanine transaminase; AST, aspartate transaminase; GGT, gamma glutamyl transferase; HbA1c, haemoglobin A1c; CRP, C-reactive protein; SFA, saturated fatty acid.

**Table S2 Distribution of plasma metabolic markers by quartiles of plasma phospholipid saturated fatty acid (SFA) groups: EPIC-InterAct Study**

|  | n | SFA groups | Q1 | Q2 | Q3 | Q4 |
| --- | --- | --- | --- | --- | --- | --- |
| TC (mmol/l) | 15304 | Odd-chain SFA | 6.11 (1.15) | 5.91 (1.11) | 5.85 (1.08) | 5.86 (1.08) |
|  |  | Even-chain SFA | 5.76 (1.06) | 5.89 (1.1) | 5.99 (1.1) | 6.09 (1.15) |
|  |  | Very-long-chain SFA | 5.81 (1.1) | 5.88 (1.1) | 5.94 (1.08) | 6.12 (1.13) |
| HDL-C (mmol/l) | 15306 | Odd-chain SFA | 1.44 (0.43) | 1.47 (0.41) | 1.52 (0.4) | 1.55 (0.43) |
|  |  | Even-chain SFA | 1.63 (0.41) | 1.52 (0.41) | 1.45 (0.41) | 1.38 (0.4) |
|  |  | Very-long-chain SFA | 1.51 (0.46) | 1.49 (0.41) | 1.49 (0.4) | 1.49 (0.39) |
| LDL-C (mmol/l) | 15125 | Odd-chain SFA | 3.92 (1.03) | 3.82 (1.01) | 3.79 (0.99) | 3.8 (0.97) |
|  |  | Even-chain SFA | 3.67 (0.97) | 3.81 (1) | 3.89 (0.99) | 3.97 (1.02) |
|  |  | Very-long-chain SFA | 3.54 (0.95) | 3.77 (0.97) | 3.91 (0.97) | 4.1 (1.02) |
| TC/ HDL-C ^§^ | 15304 | Odd-chain SFA | 4.34 (3.41-5.5) | 4.07 (3.24-5.1) | 3.86 (3.17-4.77) | 3.79 (3.11-4.73) |
|  |  | Even-chain SFA | 3.54 (2.93-4.36) | 3.92 (3.18-4.83) | 4.19 (3.36-5.25) | 4.5 (3.56-5.64) |
|  |  | Very-long-chain SFA | 3.85 (3.06-5) | 3.94 (3.18-4.92) | 4 (3.28-5) | 4.14 (3.38-5.15) |
| TG (mmol/l) ^§^ | 15303 | Odd-chain SFA | 1.4 (0.9-2) | 1.2 (0.8-1.7) | 1 (0.8-1.5) | 1 (0.7-1.3) |
|  |  | Even-chain SFA | 0.9 (0.6-1.2) | 1 (0.8-1.5) | 1.2 (0.9-1.7) | 1.4 (1-2) |
|  |  | Very-long-chain SFA | 1.3 (0.9-2) | 1.1 (0.8-1.6) | 1 (0.8-1.5) | 1 (0.7-1.4) |
| ApoA1 (µmol/l) | 15308 | Odd-chain SFA | 55.3 (10.8) | 54.4 (10.3) | 54.6 (10) | 55.2 (10.3) |
|  |  | Even-chain SFA | 56.6 (9.9) | 55.2 (10.3) | 54.2 (10.3) | 53.3 (10.7) |
|  |  | Very-long-chain SFA | 57 (11.2) | 54.7 (10.2) | 53.7 (9.9) | 53.9 (9.8) |
| ApoB (µmol/l) | 15295 | Odd-chain SFA | 2.06 (0.53) | 1.94 (0.5) | 1.9 (0.48) | 1.88 (0.46) |
|  |  | Even-chain SFA | 1.81 (0.46) | 1.92 (0.49) | 1.98 (0.5) | 2.07 (0.52) |
|  |  | Very-long-chain SFA | 1.86 (0.51) | 1.92 (0.49) | 1.96 (0.48) | 2.03 (0.49) |
| ApoB/A1 | 15295 | Odd-chain SFA | 0.04 (0.01) | 0.04 (0.01) | 0.04 (0.01) | 0.04 (0.01) |
|  |  | Even-chain SFA | 0.03 (0.01) | 0.04 (0.01) | 0.04 (0.01) | 0.04 (0.01) |
|  |  | Very-long-chain SFA | 0.03 (0.01) | 0.04 (0.01) | 0.04 (0.01) | 0.04 (0.01) |
| ALT (µkat/l) ^§^ | 15711 | Odd-chain SFA | 0.37 (0.27-0.52) | 0.32 (0.23-0.43) | 0.3 (0.23-0.4) | 0.28 (0.23-0.37) |
|  |  | Even-chain SFA | 0.28 (0.22-0.37) | 0.3 (0.23-0.4) | 0.32 (0.25-0.43) | 0.35 (0.25-0.5) |
|  |  | Very-long-chain SFA | 0.32 (0.23-0.47) | 0.32 (0.23-0.42) | 0.3 (0.23-0.4) | 0.3 (0.23-0.4) |
| AST (µkat/l) ^§^ | 15298 | Odd-chain SFA | 0.47 (0.4-0.57) | 0.45 (0.38-0.52) | 0.43 (0.38-0.5) | 0.43 (0.38-0.5) |
|  |  | Even-chain SFA | 0.43 (0.38-0.5) | 0.43 (0.38-0.52) | 0.45 (0.38-0.53) | 0.47 (0.4-0.57) |
|  |  | Very-long-chain SFA | 0.45 (0.38-0.55) | 0.45 (0.38-0.52) | 0.43 (0.38-0.52) | 0.45 (0.4-0.52) |
| GGT (µkat/l) ^§^ | 15274 | Odd-chain SFA | 0.48 (0.32-0.8) | 0.35 (0.25-0.52) | 0.3 (0.22-0.45) | 0.27 (0.22-0.38) |
|  |  | Even-chain SFA | 0.27 (0.2-0.38) | 0.32 (0.23-0.47) | 0.37 (0.27-0.58) | 0.43 (0.28-0.72) |
|  |  | Very-long-chain SFA | 0.4 (0.25-0.68) | 0.33 (0.23-0.52) | 0.32 (0.23-0.47) | 0.32 (0.23-0.47) |
| HbA1c (mmol/mol) | 15295 | Odd-chain SFA | 29 (19-48) | 21 (15-31) | 18 (13-27) | 16 (13-23) |
|  |  | Even-chain SFA | 16 (12-23) | 19 (14-28) | 22 (16-35) | 26 (17-43) |
|  |  | Very-long-chain SFA | 24 (15-41) | 20 (14-31) | 19 (14-28) | 19 (14-28) |
| CRP (nmol/l) ^§^ | 15068 | Odd-chain SFA | 12.95 (6.48-27.52) | 10.71 (5.33-23.05) | 9.43 (4.67-20.29) | 8.67 (4.19-17.43) |
|  |  | Even-chain SFA | 7.71 (3.81-15.05) | 9.52 (4.76-20) | 11.33 (5.71-24.1) | 13.81 (6.57-30.38) |
|  |  | Very-long-chain SFA | 10.86 (5.14-22.67) | 9.9 (4.76-21.62) | 9.9 (5.05-21.52) | 10.57 (5.24-22.19) |

^§^ These metabolic markers were presented as median (interquartile range), other metabolic markers were presented as mean (SD);

Grouping of SFA: odd-chain SFA (C15:0 + C17:0), even-chain SFA (C14:0 + C16:0 + C18:0), very-long-chain SFA (C20:0 + C22:0 + C23:0 + C24:0);

TC, total cholesterol; HDL-C, high-density lipoprotein cholesterol; LDL-C, low-density lipoprotein cholesterol; TG, triglycerides; ApoA1, apolipoprotein A-1; ApoB, apolipoprotein B; ALT, alanine transaminase; GGT, gamma glutamyl transferase; AST, aspartate transaminase; HbA1c, haemoglobin A1c; CRP, C-reactive protein.

**Table S3 Pairwise correlation of plasma phospholipid saturated fatty acids in the subcohort of EPIC-InterAct study** ^δ^

|  | C15:0 | C17:0 | Odd-chain SFA | C14:0 | C16:0 | C18:0 | Even-chain SFA | C20:0 | C22:0 | C23:0 | C24:0 | Very-long-chain SFA |
| --- | --- | --- | --- | --- | --- | --- | --- | --- | --- | --- | --- | --- |
| C15:0 | 1 |  |  |  |  |  |  |  |  |  |  |  |
| C17:0 | 0.50 | 1 |  |  |  |  |  |  |  |  |  |  |
| Odd-chain SFA | 0.80 | 0.90 | 1 |  |  |  |  |  |  |  |  |  |
| C14:0 | 0.46 | -0.12 | 0.14 | 1 |  |  |  |  |  |  |  |  |
| C16:0 | 0.05 | -0.39 | -0.23 | 0.34 | 1 |  |  |  |  |  |  |  |
| C18:0 | -0.16 | 0.25 | 0.08 | -0.23 | -0.70 | 1 |  |  |  |  |  |  |
| Even-chain SFA | -0.08 | -0.32 | -0.25 | 0.31 | 0.62 | 0.05 | 1.00 |  |  |  |  |  |
| C20:0 | 0.14 | 0.22 | 0.21 | -0.09 | -0.22 | 0.13 | -0.19 | 1 |  |  |  |  |
| C22:0 | 0.19 | 0.23 | 0.24 | -0.11 | -0.23 | 0.19 | -0.15 | 0.51 | 1 |  |  |  |
| C23:0 | 0.38 | 0.37 | 0.42 | 0.05 | -0.19 | 0.10 | -0.17 | 0.35 | 0.57 | 1 |  |  |
| C24:0 | 0.13 | 0.17 | 0.18 | -0.09 | -0.16 | 0.13 | -0.10 | 0.38 | 0.75 | 0.56 | 1 |  |
| Very-long-chain SFA | 0.24 | 0.28 | 0.30 | -0.08 | -0.24 | 0.17 | -0.18 | 0.63 | 0.90 | 0.74 | 0.85 | 1 |

^δ^ Pairwise Spearman correlation coefficients were calculated in the subcohort (n=15919).

**Table S4 Association of plasma phospholipid odd-chain saturated fatty acids (per 1 SD difference) with metabolic markers: EPIC-InterAct Study**

|  |  | C15:0 |  | C17:0 |  | Odd-chain SFA |  |
| --- | --- | --- | --- | --- | --- | --- | --- |
|  | Model | Standardised Difference (CI) ^δ^ | I^2^, % | Standardised Difference (CI) ^δ^ | I^2^, % | Standardised Difference (CI) ^δ^ | I^2^, % |
| TC (mmol/l) | Model 1 | -0.087(-0.151, -0.022) | 79 | -0.106(-0.162, -0.05) | 73 | -0.107(-0.163, -0.051) | 74 |
|  | Model 2 | -0.081(-0.146, -0.015) | 79 | -0.093(-0.159, -0.028) | 80 | -0.096(-0.16, -0.033) | 80 |
|  | Model 3 | -0.064(-0.123, -0.006)* | 69 | -0.079(-0.149, -0.008)* | 79 | -0.082(-0.15, -0.014)* | 78 |
| HDL-C (mmol/l) | Model 1 | -0.027(-0.075, 0.021) | 64 | -0.003(-0.031, 0.024) | 3 | -0.015(-0.042, 0.012) | 0 |
|  | Model 2 | -0.053(-0.094, -0.012) | 54 | -0.05(-0.08, -0.02) | 17 | -0.057(-0.083, -0.031) | 0 |
|  | Model 3 | -0.027(-0.069, 0.014) | 46 | -0.014(-0.042, 0.014) | 0 | -0.023(-0.052, 0.005) | 0 |
| LDL-C (mmol/l) | Model 1 | -0.041(-0.081, -0.002) | 42 | -0.037(-0.09, 0.015) | 67 | -0.044(-0.088, 0) | 56 |
|  | Model 2 | -0.033(-0.073, 0.008) | 45 | -0.017(-0.082, 0.047) | 79 | -0.027(-0.081, 0.028) | 71 |
|  | Model 3 | -0.028(-0.066, 0.01) | 25 | -0.014(-0.09, 0.061) | 81 | -0.023(-0.085, 0.04) | 73 |
| TC/ HDL-C ^§^ | Model 1 | -0.025(-0.069, 0.02) | 59 | -0.065(-0.091, -0.038) | 0 | -0.054(-0.082, -0.025) | 12 |
|  | Model 2 | 0.002(-0.028, 0.031) | 17 | -0.015(-0.051, 0.022) | 44 | -0.012(-0.041, 0.018) | 20 |
|  | Model 3 | -0.009(-0.039, 0.02) | 4 | -0.039(-0.076, -0.003)* | 37 | -0.033(-0.063, -0.003)* | 11 |
| TG (mmol/l) ^§^ | Model 1 | -0.101(-0.169, -0.033) | 83 | -0.208(-0.27, -0.145) | 81 | -0.181(-0.242, -0.121) | 81 |
|  | Model 2 | -0.076(-0.134, -0.018) | 78 | -0.165(-0.223, -0.108) | 78 | -0.143(-0.2, -0.085) | 79 |
|  | Model 3 | -0.073(-0.121, -0.025)* | 61 | -0.18(-0.234, -0.126)* | 72 | -0.156(-0.209, -0.103)* | 70 |
| ApoA1 (µmol/l) | Model 1 | -0.096(-0.165, -0.026) | 83 | -0.143(-0.202, -0.084) | 78 | -0.133(-0.181, -0.085) | 67 |
|  | Model 2 | -0.113(-0.183, -0.043) | 84 | -0.178(-0.248, -0.109) | 84 | -0.163(-0.211, -0.114) | 68 |
|  | Model 3 | -0.076(-0.13, -0.022)* | 67 | -0.137(-0.195, -0.079)* | 73 | -0.125(-0.169, -0.081)* | 53 |
| ApoB (µmol/l) | Model 1 | -0.087(-0.136, -0.037) | 65 | -0.106(-0.149, -0.063) | 55 | -0.109(-0.15, -0.069) | 52 |
|  | Model 2 | -0.073(-0.121, -0.024) | 64 | -0.077(-0.134, -0.021) | 74 | -0.084(-0.135, -0.032) | 70 |
|  | Model 3 | -0.063(-0.104, -0.021)* | 40 | -0.072(-0.129, -0.015)* | 70 | -0.079(-0.128, -0.029)* | 60 |
| ApoB/A1 | Model 1 | -0.013(-0.041, 0.016) | 0 | -0.017(-0.044, 0.01) | 0 | -0.017(-0.045, 0.01) | 0 |
|  | Model 2 | 0.006(-0.022, 0.034) | 0 | 0.028(-0.016, 0.073) | 60 | 0.016(-0.013, 0.045) | 11 |
|  | Model 3 | -0.002(-0.032, 0.028) | 0 | 0.012(-0.033, 0.057) | 54 | 0.002(-0.027, 0.032) | 0 |
| ALT (µkat/l) ^§^ | Model 1 | -0.077(-0.132, -0.021) | 73 | -0.124(-0.186, -0.063) | 79 | -0.115(-0.17, -0.061) | 74 |
|  | Model 2 | -0.052(-0.098, -0.007) | 62 | -0.08(-0.128, -0.032) | 67 | -0.076(-0.121, -0.031) | 63 |
|  | Model 3 | -0.049(-0.088, -0.011)* | 36 | -0.083(-0.132, -0.034)* | 61 | -0.079(-0.121, -0.037)* | 48 |
| AST (µkat/l) ^§^ | Model 1 | -0.105(-0.194, -0.016) | 89 | -0.135(-0.216, -0.054) | 87 | -0.132(-0.21, -0.053) | 87 |
|  | Model 2 | -0.094(-0.177, -0.01) | 88 | -0.116(-0.191, -0.042) | 84 | -0.115(-0.188, -0.042) | 85 |
|  | Model 3 | -0.097(-0.174, -0.019)* | 82 | -0.124(-0.196, -0.052)* | 80 | -0.125(-0.192, -0.059)* | 77 |
| GGT (µkat/l) ^§^ | Model 1 | -0.198(-0.335, -0.06) | 96 | -0.258(-0.388, -0.128) | 96 | -0.251(-0.38, -0.123) | 96 |
|  | Model 2 | -0.18(-0.31, -0.05) | 96 | -0.23(-0.353, -0.106) | 96 | -0.226(-0.348, -0.103) | 96 |
|  | Model 3 | -0.139(-0.245, -0.032)* | 93 | -0.196(-0.303, -0.09)* | 93 | -0.193(-0.301, -0.085)* | 93 |
| HbA1c (mmol/mol) | Model 1 | 0.01(-0.025, 0.045) | 36 | -0.039(-0.073, -0.006) | 33 | -0.021(-0.052, 0.01) | 23 |
|  | Model 2 | 0.027(-0.001, 0.054) | 0 | -0.013(-0.051, 0.026) | 49 | 0.004(-0.027, 0.035) | 24 |
|  | Model 3 | 0.015(-0.015, 0.045) | 0 | -0.032(-0.068, 0.004) | 33 | -0.015(-0.045, 0.014) | 3 |
| CRP (nmol/l) ^§^ | Model 1 | -0.082(-0.163, -0.002) | 86 | -0.177(-0.234, -0.119) | 73 | -0.149(-0.21, -0.089) | 76 |
|  | Model 2 | -0.051(-0.116, 0.014) | 81 | -0.118(-0.157, -0.079) | 47 | -0.097(-0.145, -0.05) | 65 |
|  | Model 3 | -0.043(-0.101, 0.015) | 70 | -0.12(-0.158, -0.082)* | 32 | -0.1(-0.144, -0.055)* | 51 |

^δ^ Values represent standardised difference (in SD unit of the metabolic marker) per 1 SD of each saturated fatty acid (SFA). The confidence intervals were corrected for multiple testing based on a familywise error rate of 5%. * indicated that the p-value for the association <0.00068;

^§^ TG, TC/ HDL-C, ALT, AST, GGT and CRP were log-transformed;

Model 1, adjusted for age, sex and centre;

Model 2, model 1 plus BMI;

Model 3, model 2 plus physical activity, smoking status, alcohol drinking, educational level and total energy intake;

All the analyses were conducted in each country and then pooled by random-effects meta-analysis. I^2^ represent heterogeneity across the countries;

TC, total cholesterol; HDL-C, high-density lipoprotein cholesterol; LDL-C, low-density lipoprotein cholesterol; TG, triglycerides; ApoA1, apolipoprotein A-1; ApoB, apolipoprotein B; ALT, alanine transaminase; GGT, gamma glutamyl transferase; AST, aspartate transaminase; HbA1c, haemoglobin A1c; CRP, C-reactive protein.

**Table S5 Association of plasma phospholipid even-chain saturated fatty acids (per 1 SD difference) with metabolic markers: EPIC-InterAct Study**

*

|  |  | C14:0 | | C16:0 | | C18:0 | | Even-chain SFA | |
| --- | --- | --- | --- | --- | --- | --- | --- | --- | --- |
|  | Model | Standardised Difference (CI) ^δ^ | I^2^, % | Standardised Difference (CI) ^δ^ | I^2^, % | Standardised Difference (CI) ^δ^ | I^2^, % | Standardised Difference (CI) ^δ^ | I^2^, % |
| TC (mmol/l) | Model 1 | 0.05(-0.018, 0.118) | 81 | -0.012(-0.098, 0.074) | 89 | 0.066(-0.013, 0.145) | 87 | 0.068(0.012, 0.123) | 74 |
|  | Model 2 | 0.044(-0.024, 0.112) | 81 | -0.014(-0.1, 0.073) | 89 | 0.055(-0.021, 0.13) | 86 | 0.052(-0.008, 0.111) | 76 |
|  | Model 3 | 0.047(-0.021, 0.115) | 80 | -0.031(-0.117, 0.056) | 87 | 0.072(0, 0.144) | 83 | 0.047(-0.009, 0.103) | 71 |
| HDL-C (mmol/l) | Model 1 | -0.013(-0.065, 0.038) | 69 | -0.003(-0.044, 0.039) | 54 | -0.18(-0.227, -0.133) | 67 | -0.217(-0.32, -0.115) | 93 |
|  | Model 2 | 0.005(-0.035, 0.045) | 51 | -0.001(-0.03, 0.029) | 17 | -0.137(-0.173, -0.101) | 45 | -0.163(-0.242, -0.084) | 89 |
|  | Model 3 | -0.008(-0.042, 0.025) | 29 | -0.049(-0.077, -0.022)* | 0 | -0.102(-0.147, -0.057)* | 63 | -0.194(-0.276, -0.111)* | 89 |
| LDL-C (mmol/l) | Model 1 | 0.007(-0.069, 0.083) | 84 | -0.032(-0.113, 0.048) | 86 | 0.111(0.037, 0.185) | 85 | 0.084(0.03, 0.139) | 72 |
|  | Model 2 | -0.001(-0.076, 0.074) | 84 | -0.035(-0.115, 0.046) | 87 | 0.096(0.024, 0.168) | 84 | 0.063(0.006, 0.119) | 73 |
|  | Model 3 | 0.005(-0.068, 0.078) | 82 | -0.034(-0.114, 0.046) | 85 | 0.101(0.033, 0.168)* | 80 | 0.069(0.015, 0.124)* | 68 |
| TC/ HDL-C ^§^ | Model 1 | 0.044(-0.02, 0.107) | 80 | 0(-0.064, 0.065) | 81 | 0.186(0.119, 0.252) | 84 | 0.225(0.11, 0.34) | 95 |
|  | Model 2 | 0.025(-0.03, 0.079) | 75 | -0.002(-0.058, 0.054) | 77 | 0.141(0.093, 0.19) | 71 | 0.169(0.076, 0.262) | 92 |
|  | Model 3 | 0.037(-0.011, 0.085) | 67 | 0.03(-0.019, 0.08) | 68 | 0.123(0.078, 0.168)* | 65 | 0.19(0.099, 0.282)* | 92 |
| TG (mmol/l) ^§^ | Model 1 | 0.153(0.084, 0.222) | 84 | 0.079(-0.001, 0.158) | 88 | 0.096(0.037, 0.154) | 79 | 0.238(0.108, 0.368) | 96 |
|  | Model 2 | 0.136(0.078, 0.195) | 79 | 0.077(0.001, 0.153) | 88 | 0.052(0.01, 0.093) | 60 | 0.186(0.072, 0.3) | 95 |
|  | Model 3 | 0.146(0.093, 0.2)* | 74 | 0.089(0.022, 0.155)* | 82 | 0.047(0.001, 0.092)* | 64 | 0.19(0.084, 0.296)* | 94 |
| ApoA1 (µmol/l) | Model 1 | 0.078(0.038, 0.118) | 49 | 0.076(0.027, 0.124) | 66 | -0.181(-0.233, -0.128) | 74 | -0.104(-0.171, -0.037) | 83 |
|  | Model 2 | 0.089(0.051, 0.127) | 46 | 0.077(0.035, 0.119) | 57 | -0.157(-0.206, -0.109) | 69 | -0.07(-0.122, -0.018) | 71 |
|  | Model 3 | 0.079(0.046, 0.112)* | 25 | 0.022(-0.021, 0.064) | 51 | -0.119(-0.169, -0.069)* | 69 | -0.106(-0.167, -0.046)* | 77 |
| ApoB (µmol/l) | Model 1 | 0.032(-0.038, 0.103) | 83 | 0.033(-0.044, 0.111) | 86 | 0.091(0.015, 0.166) | 86 | 0.154(0.078, 0.23) | 87 |
|  | Model 2 | 0.02(-0.048, 0.088) | 82 | 0.03(-0.045, 0.106) | 86 | 0.065(-0.003, 0.134) | 83 | 0.122(0.05, 0.193) | 84 |
|  | Model 3 | 0.025(-0.039, 0.09) | 79 | 0.03(-0.04, 0.1) | 81 | 0.07(0.01, 0.131)* | 77 | 0.123(0.058, 0.188)* | 79 |
| ApoB/A1 | Model 1 | -0.012(-0.066, 0.042) | 71 | 0.003(-0.053, 0.059) | 74 | 0.152(0.088, 0.216) | 82 | 0.183(0.087, 0.278) | 92 |
|  | Model 2 | -0.027(-0.077, 0.022) | 67 | 0(-0.049, 0.049) | 68 | 0.118(0.067, 0.17) | 72 | 0.139(0.061, 0.217) | 88 |
|  | Model 3 | -0.017(-0.058, 0.023) | 49 | 0.029(-0.018, 0.076) | 60 | 0.101(0.057, 0.146)* | 60 | 0.158(0.081, 0.235)* | 87 |
| ALT (µkat/l) ^§^ | Model 1 | 0.074(0.024, 0.125) | 68 | -0.014(-0.065, 0.037) | 69 | 0.139(0.071, 0.207) | 84 | 0.155(0.067, 0.242) | 90 |
|  | Model 2 | 0.057(0.023, 0.091) | 34 | -0.015(-0.055, 0.026) | 54 | 0.096(0.042, 0.149) | 75 | 0.099(0.037, 0.161) | 81 |
|  | Model 3 | 0.056(0.018, 0.093)* | 39 | -0.024(-0.056, 0.007) | 15 | 0.109(0.058, 0.161)* | 70 | 0.099(0.041, 0.157)* | 76 |
| AST (µkat/l) ^§^ | Model 1 | 0.044(0.005, 0.083) | 42 | -0.001(-0.034, 0.031) | 19 | 0.063(0.008, 0.117) | 73 | 0.076(0.016, 0.137) | 78 |
|  | Model 2 | 0.036(0.002, 0.069) | 22 | -0.001(-0.03, 0.027) | 0 | 0.043(-0.005, 0.09) | 64 | 0.05(0.002, 0.099) | 65 |
|  | Model 3 | 0.032(-0.002, 0.067) | 22 | -0.011(-0.042, 0.02) | 0 | 0.054(0.003, 0.106)* | 65 | 0.05(0.008, 0.093)* | 48 |
| GGT (µkat/l) ^§^ | Model 1 | 0.075(-0.01, 0.16) | 90 | 0.073(-0.008, 0.154) | 89 | 0.067(0.014, 0.121) | 76 | 0.196(0.068, 0.325) | 96 |
|  | Model 2 | 0.062(-0.01, 0.134) | 86 | 0.073(-0.004, 0.149) | 88 | 0.033(-0.012, 0.077) | 65 | 0.159(0.044, 0.273) | 95 |
|  | Model 3 | 0.063(-0.004, 0.131) | 83 | 0.023(-0.037, 0.083) | 78 | 0.07(0.027, 0.113)* | 60 | 0.124(0.031, 0.218)* | 92 |
| HbA1c (mmol/mol) | Model 1 | 0.042(-0.018, 0.102) | 78 | -0.032(-0.08, 0.016) | 66 | 0.089(0.057, 0.12) | 26 | 0.063(0.01, 0.117) | 75 |
|  | Model 2 | 0.03(-0.02, 0.081) | 70 | -0.033(-0.08, 0.013) | 65 | 0.063(0.035, 0.092) | 13 | 0.03(-0.014, 0.074) | 62 |
|  | Model 3 | 0.035(-0.008, 0.078) | 56 | -0.013(-0.055, 0.029) | 52 | 0.042(0.015, 0.07)* | 0 | 0.036(-0.006, 0.077) | 54 |
| CRP (nmol/l) ^§^ | Model 1 | 0.031(-0.045, 0.108) | 84 | 0.149(0.105, 0.194) | 55 | 0.017(-0.053, 0.087) | 83 | 0.223(0.128, 0.319) | 91 |
|  | Model 2 | 0.006(-0.051, 0.062) | 74 | 0.148(0.11, 0.187) | 48 | -0.049(-0.113, 0.014) | 81 | 0.151(0.088, 0.213) | 80 |
|  | Model 3 | 0.017(-0.034, 0.068) | 66 | 0.165(0.116, 0.214)* | 63 | -0.059(-0.129, 0.01) | 83 | 0.15(0.096, 0.205)* | 71 |

^δ^ Values represent standardised difference (in SD unit of the metabolic marker) per 1 SD of each saturated fatty acid (SFA). The confidence intervals were corrected for multiple testing based on a familywise error rate of 5%. * indicated that the p-value for the association <0.00068;

^§^ TG, TC/ HDL-C, CRP, ALT, AST and GGT were log-transformed;

Model 1, adjusted for age, sex and centre;

Model 2, model 1 plus BMI;

Model 3, model 2 plus physical activity, smoking status, alcohol drinking, educational level and total energy intake;

All the analyses were conducted in each country and then pooled by random-effects meta-analysis. I^2^ represent heterogeneity across the countries;

TC, total cholesterol; HDL-C, high-density lipoprotein cholesterol; LDL-C, low-density lipoprotein cholesterol; TG, triglycerides; ApoA1, apolipoprotein A-1; ApoB, apolipoprotein B; ALT, alanine transaminase; GGT, gamma glutamyl transferase; AST, aspartate transaminase; HbA1c, haemoglobin A1c; CRP, C-reactive protein.

**Table S6 Association of plasma phospholipid very-long-chain saturated fatty acids (per 1 SD difference) with metabolic markers: EPIC-InterAct Study**

|  |  | C20:0 |  | C22:0 |  | C23:0 |  | C24:0 |  | Very-long-chain SFA |  |
| --- | --- | --- | --- | --- | --- | --- | --- | --- | --- | --- | --- |
|  | Model | Standardised Difference (CI) ^δ^ | I^2^, % | Standardised Difference (CI) ^δ^ | I^2^,% | Standardised Difference (CI) ^δ^ | I^2^,% | Standardised Difference (CI) ^δ^ | I^2^, % | Standardised Difference (CI) ^δ^ | I^2^, % |
| TC (mmol/l) | Model 1 | -0.028(-0.069, 0.014) | 53 | 0.077(0.019, 0.135) | 74 | 0.083(0.033, 0.133) | 68 | 0.091(0.032, 0.149) | 76 | 0.076(0.027, 0.126) | 64 |
|  | Model 2 | -0.024(-0.066, 0.018) | 54 | 0.075(0.017, 0.132) | 73 | 0.083(0.034, 0.132) | 66 | 0.096(0.034, 0.158) | 78 | 0.079(0.028, 0.129) | 66 |
|  | Model 3 | -0.019(-0.062, 0.025) | 54 | 0.086(0.025, 0.146)* | 74 | 0.098(0.046, 0.151)* | 68 | 0.104(0.037, 0.171)* | 80 | 0.092(0.035, 0.15)* | 71 |
| HDL-C (mmol/l) | Model 1 | -0.035(-0.087, 0.016) | 72 | -0.063(-0.115, -0.011) | 69 | -0.01(-0.037, 0.016) | 0 | -0.006(-0.032, 0.021) | 0 | -0.036(-0.079, 0.006) | 55 |
|  | Model 2 | -0.045(-0.097, 0.006) | 74 | -0.057(-0.105, -0.008) | 67 | -0.013(-0.039, 0.012) | 0 | -0.016(-0.044, 0.012) | 14 | -0.042(-0.085, 0.002) | 60 |
|  | Model 3 | -0.017(-0.056, 0.023) | 54 | -0.032(-0.071, 0.006) | 47 | -0.004(-0.03, 0.023) | 0 | -0.011(-0.037, 0.015) | 0 | -0.02(-0.052, 0.011) | 24 |
| LDL-C (mmol/l) | Model 1 | 0.048(-0.021, 0.117) | 83 | 0.188(0.078, 0.298) | 93 | 0.142(0.057, 0.228) | 89 | 0.186(0.071, 0.302) | 94 | 0.191(0.075, 0.307) | 94 |
|  | Model 2 | 0.052(-0.019, 0.122) | 84 | 0.184(0.075, 0.292) | 93 | 0.142(0.059, 0.226) | 89 | 0.192(0.074, 0.311) | 94 | 0.192(0.075, 0.31) | 94 |
|  | Model 3 | 0.048(-0.021, 0.116) | 81 | 0.187(0.074, 0.3)* | 93 | 0.15(0.063, 0.237)* | 88 | 0.195(0.073, 0.318)* | 94 | 0.198(0.074, 0.323)* | 94 |
| TC/ HDL-C ^§^ | Model 1 | 0.007(-0.051, 0.065) | 79 | 0.088(0.025, 0.151) | 80 | 0.054(0.012, 0.096) | 58 | 0.044(0.007, 0.081) | 43 | 0.066(0.014, 0.118) | 71 |
|  | Model 2 | 0.018(-0.039, 0.075) | 79 | 0.08(0.022, 0.138) | 78 | 0.055(0.02, 0.09) | 45 | 0.059(0.016, 0.102) | 62 | 0.071(0.019, 0.123) | 73 |
|  | Model 3 | -0.003(-0.044, 0.037) | 57 | 0.065(0.011, 0.118)* | 72 | 0.055(0.016, 0.093)* | 52 | 0.057(0.013, 0.102)* | 64 | 0.06(0.012, 0.108)* | 66 |
| TG (mmol/l) ^§^ | Model 1 | -0.155(-0.244, -0.066) | 91 | -0.162(-0.292, -0.032) | 96 | -0.103(-0.18, -0.026) | 88 | -0.191(-0.319, -0.062) | 96 | -0.196(-0.343, -0.05) | 97 |
|  | Model 2 | -0.146(-0.225, -0.068) | 89 | -0.171(-0.302, -0.041) | 96 | -0.102(-0.18, -0.024) | 89 | -0.178(-0.296, -0.06) | 95 | -0.193(-0.334, -0.052) | 97 |
|  | Model 3 | -0.153(-0.229, -0.078)* | 88 | -0.181(-0.306, -0.056)* | 95 | -0.098(-0.166, -0.03)* | 84 | -0.179(-0.289, -0.069)* | 94 | -0.2(-0.334, -0.066)* | 96 |
| ApoA1 (µmol/l) | Model 1 | -0.118(-0.183, -0.054) | 83 | -0.159(-0.264, -0.053) | 93 | -0.065(-0.111, -0.018) | 66 | -0.104(-0.182, -0.026) | 88 | -0.148(-0.249, -0.046) | 93 |
|  | Model 2 | -0.126(-0.195, -0.057) | 85 | -0.156(-0.261, -0.051) | 93 | -0.067(-0.113, -0.021) | 66 | -0.112(-0.195, -0.029) | 89 | -0.151(-0.255, -0.047) | 93 |
|  | Model 3 | -0.095(-0.153, -0.036)* | 79 | -0.128(-0.223, -0.034)* | 91 | -0.049(-0.087, -0.011)* | 46 | -0.103(-0.178, -0.028)* | 86 | -0.126(-0.219, -0.034)* | 91 |
| ApoB (µmol/l) | Model 1 | 0.007(-0.05, 0.064) | 76 | 0.121(0.04, 0.202) | 87 | 0.087(0.026, 0.147) | 79 | 0.108(0.035, 0.18) | 85 | 0.111(0.036, 0.186) | 85 |
|  | Model 2 | 0.014(-0.045, 0.073) | 78 | 0.116(0.038, 0.194) | 86 | 0.087(0.03, 0.145) | 77 | 0.117(0.039, 0.195) | 87 | 0.114(0.037, 0.192) | 86 |
|  | Model 3 | 0.012(-0.041, 0.065) | 70 | 0.121(0.039, 0.204)* | 87 | 0.1(0.034, 0.167)* | 81 | 0.123(0.039, 0.207)* | 88 | 0.123(0.038, 0.208)* | 88 |
| ApoB/A1 | Model 1 | 0.062(-0.005, 0.129) | 83 | 0.174(0.066, 0.282) | 93 | 0.098(0.03, 0.166) | 84 | 0.131(0.044, 0.218) | 90 | 0.157(0.055, 0.26) | 92 |
|  | Model 2 | 0.071(0, 0.142) | 85 | 0.168(0.063, 0.273) | 93 | 0.099(0.035, 0.163) | 83 | 0.143(0.05, 0.235) | 91 | 0.162(0.057, 0.266) | 93 |
|  | Model 3 | 0.053(-0.006, 0.111) | 78 | 0.157(0.051, 0.263)* | 93 | 0.099(0.03, 0.169)* | 84 | 0.142(0.046, 0.237)* | 91 | 0.154(0.047, 0.261)* | 93 |
| ALT (µkat/l) ^§^ | Model 1 | -0.047(-0.099, 0.004) | 72 | -0.032(-0.094, 0.03) | 78 | -0.016(-0.06, 0.028) | 61 | -0.044(-0.104, 0.015) | 77 | -0.044(-0.112, 0.024) | 82 |
|  | Model 2 | -0.039(-0.085, 0.007) | 66 | -0.04(-0.1, 0.02) | 79 | -0.014(-0.059, 0.03) | 64 | -0.032(-0.082, 0.018) | 70 | -0.041(-0.104, 0.022) | 80 |
|  | Model 3 | -0.035(-0.07, 0.001) | 39 | -0.036(-0.086, 0.014) | 66 | -0.011(-0.056, 0.033) | 60 | -0.029(-0.076, 0.018) | 63 | -0.036(-0.088, 0.017) | 69 |
| AST (µkat/l) ^§^ | Model 1 | -0.056(-0.118, 0.006) | 80 | -0.07(-0.155, 0.014) | 88 | -0.029(-0.079, 0.021) | 68 | -0.067(-0.148, 0.015) | 88 | -0.074(-0.166, 0.018) | 90 |
|  | Model 2 | -0.052(-0.11, 0.007) | 77 | -0.074(-0.16, 0.012) | 89 | -0.028(-0.078, 0.021) | 68 | -0.06(-0.137, 0.016) | 86 | -0.073(-0.163, 0.017) | 90 |
|  | Model 3 | -0.047(-0.098, 0.004) | 67 | -0.069(-0.145, 0.007) | 84 | -0.024(-0.067, 0.02) | 53 | -0.057(-0.129, 0.015) | 83 | -0.068(-0.148, 0.013) | 86 |
| GGT (µkat/l) ^§^ | Model 1 | -0.099(-0.185, -0.014) | 91 | -0.108(-0.22, 0.003) | 94 | -0.099(-0.189, -0.008) | 92 | -0.116(-0.223, -0.008) | 94 | -0.137(-0.268, -0.007) | 96 |
|  | Model 2 | -0.092(-0.169, -0.015) | 89 | -0.115(-0.229, -0.001) | 95 | -0.098(-0.189, -0.007) | 92 | -0.106(-0.206, -0.006) | 93 | -0.135(-0.263, -0.007) | 96 |
|  | Model 3 | -0.071(-0.128, -0.015)* | 79 | -0.094(-0.187, -0.001)* | 91 | -0.072(-0.135, -0.009)* | 82 | -0.092(-0.178, -0.007)* | 90 | -0.111(-0.213, -0.009)* | 93 |
| HbA1c (mmol/mol) | Model 1 | 0.001(-0.046, 0.048) | 67 | 0.053(0.005, 0.101) | 65 | 0.053(0.011, 0.095) | 59 | 0.027(-0.015, 0.069) | 56 | 0.044(-0.002, 0.089) | 61 |
|  | Model 2 | 0.008(-0.037, 0.054) | 66 | 0.049(0.004, 0.093) | 60 | 0.054(0.012, 0.096) | 60 | 0.034(-0.01, 0.078) | 61 | 0.047(0.001, 0.092) | 62 |
|  | Model 3 | -0.009(-0.038, 0.019) | 13 | 0.033(-0.009, 0.075) | 54 | 0.041(0.008, 0.073)* | 29 | 0.027(-0.013, 0.067) | 51 | 0.032(-0.009, 0.072) | 50 |
| CRP (nmol/l) ^§^ | Model 1 | -0.034(-0.082, 0.013) | 62 | 0.026(-0.006, 0.057) | 13 | -0.01(-0.06, 0.039) | 65 | -0.014(-0.059, 0.032) | 56 | -0.003(-0.038, 0.032) | 28 |
|  | Model 2 | -0.022(-0.058, 0.013) | 41 | 0.017(-0.01, 0.044) | 0 | -0.01(-0.057, 0.038) | 66 | 0.002(-0.034, 0.037) | 38 | 0.002(-0.027, 0.031) | 9 |
|  | Model 3 | -0.025(-0.054, 0.005) | 13 | 0.018(-0.015, 0.051) | 20 | 0(-0.042, 0.041) | 52 | 0.005(-0.032, 0.042) | 39 | 0.004(-0.028, 0.036) | 17 |

^δ^ Values represent standardised difference (in SD unit of the metabolic marker) per 1 SD of each saturated fatty acid (SFA).The confidence intervals were corrected for multiple testing based on a familywise error rate of 5%. * indicated that the p-value for the association <0.00068;

^§^ TG, TC/ HDL-C, ALT, AST, GGT and CRP were log-transformed;

Model 1, adjusted for age, sex and centre;

Model 2, model 1 plus BMI;

Model 3, model 2 plus physical activity, smoking status, alcohol drinking, educational level and total energy intake;

All the analyses were conducted in each country and then pooled by random-effects meta-analysis. I^2^ represent heterogeneity across the countries.

TC, total cholesterol; HDL-C, high-density lipoprotein cholesterol; LDL-C, low-density lipoprotein cholesterol; TG, triglycerides; ApoA1, apolipoprotein A-1; ApoB, apolipoprotein B; ALT, alanine transaminase; GGT, gamma glutamyl transferase; AST, aspartate transaminase; HbA1c, haemoglobin A1c; CRP, C-reactive protein.

**Table S7 Sensitivity analysis for the association of plasma phospholipid saturated fatty acid groups and metabolic markers: EPIC-InterAct Study**

|  |  |  | Odd-chain SFA |  | Even-chain SFA |  | Very-long-chain SFA |  |
| --- | --- | --- | --- | --- | --- | --- | --- | --- |
| Marker | Model | n | Standardised Difference (CI) ^δ^ | I^2^, % | Standardised Difference (CI) ^δ^ | I^2^, % | Standardised Difference (CI) ^δ^ | I^2^, % |
| TC (mmol/l) | Model 3 | 14629 | -0.082(-0.15, -0.014) | 78 | 0.047(-0.009, 0.103) | 71 | 0.092(0.035, 0.15) | 71 |
|  | Model 3a | 12560 | -0.057(-0.108, -0.005) | 59 | 0.033(-0.009, 0.074) | 44 | 0.102(0.038, 0.167) | 75 |
|  | Model 3b | 12110 | -0.065(-0.131, 0.002) | 75 | 0.037(-0.011, 0.086) | 57 | 0.078(0.028, 0.129) | 60 |
|  | Model 3c | 14629 | -0.08(-0.148, -0.012) | 77 | 0.049(-0.008, 0.106) | 71 | 0.095(0.038, 0.152) | 70 |
|  | Model 3d | 13855 | -0.083(-0.15, -0.016) | 76 | 0.046(-0.007, 0.1) | 66 | 0.094(0.035, 0.154) | 72 |
|  | Model 3e | 14279 | -0.081(-0.147, -0.015) | 76 | 0.047(-0.007, 0.101) | 68 | 0.094(0.034, 0.153) | 73 |
|  | Model 3f | 14599 | -0.082(-0.15, -0.013) | 78 | 0.046(-0.01, 0.103) | 71 | 0.092(0.034, 0.149) | 71 |
|  | Model 3g | 14629 | -0.096(-0.163, -0.029) | 76 | 0.043(-0.008, 0.094) | 64 | 0.118(0.045, 0.191) | 82 |
|  | Model 3h | 13883 | -0.074(-0.136, -0.012) | 72 | 0.046(-0.008, 0.101) | 68 | 0.097(0.037, 0.157) | 73 |
|  | Model 3i | 14171 | -0.081(-0.15, -0.012) | 78 | 0.049(-0.007, 0.104) | 69 | 0.094(0.037, 0.151) | 70 |
|  | Model 3j | 13751 | -0.08(-0.146, -0.014) | 76 | 0.041(-0.025, 0.107) | 78 | 0.097(0.036, 0.158) | 73 |
| HDL-C (mmol/l) | Model 3 | 14631 | -0.023(-0.052, 0.005) | 0 | -0.194(-0.276, -0.111) | 89 | -0.02(-0.052, 0.011) | 24 |
|  | Model 3a | 12560 | -0.034(-0.064, -0.003) | 0 | -0.184(-0.259, -0.11) | 84 | -0.026(-0.056, 0.003) | 0 |
|  | Model 3b | 12112 | -0.028(-0.059, 0.003) | 0 | -0.195(-0.29, -0.1) | 90 | -0.014(-0.044, 0.016) | 11 |
|  | Model 3c | 14631 | -0.026(-0.055, 0.003) | 0 | -0.191(-0.276, -0.106) | 89 | -0.022(-0.054, 0.01) | 26 |
|  | Model 3d | 13857 | -0.023(-0.053, 0.006) | 0 | -0.189(-0.27, -0.107) | 88 | -0.019(-0.049, 0.011) | 15 |
|  | Model 3e | 14281 | -0.026(-0.055, 0.003) | 0 | -0.193(-0.278, -0.109) | 89 | -0.021(-0.054, 0.012) | 27 |
|  | Model 3f | 14601 | -0.024(-0.053, 0.005) | 0 | -0.193(-0.275, -0.111) | 89 | -0.02(-0.051, 0.011) | 20 |
|  | Model 3g | 14631 | -0.047(-0.089, -0.004) | 50 | -0.208(-0.299, -0.116) | 91 | -0.04(-0.085, 0.005) | 60 |
|  | Model 3h | 13885 | -0.015(-0.045, 0.014) | 0 | -0.196(-0.278, -0.115) | 88 | -0.017(-0.049, 0.016) | 27 |
|  | Model 3i | 14173 | -0.022(-0.051, 0.007) | 0 | -0.192(-0.272, -0.113) | 88 | -0.021(-0.053, 0.011) | 25 |
|  | Model 3j | 13753 | -0.03(-0.059, -0.001) | 0 | -0.182(-0.265, -0.098) | 89 | -0.02(-0.051, 0.011) | 18 |
| LDL-C (mmol/l) | Model 3 | 14460 | -0.023(-0.085, 0.04) | 73 | 0.069(0.015, 0.124) | 68 | 0.198(0.074, 0.323) | 94 |
|  | Model 3a | 12448 | 0.003(-0.047, 0.053) | 56 | 0.058(0.014, 0.102) | 48 | 0.204(0.085, 0.323) | 93 |
|  | Model 3b | 11963 | -0.006(-0.067, 0.055) | 69 | 0.062(0.02, 0.104) | 42 | 0.182(0.059, 0.304) | 93 |
|  | Model 3c | 14460 | -0.018(-0.083, 0.047) | 74 | 0.071(0.016, 0.125) | 68 | 0.201(0.077, 0.326) | 94 |
|  | Model 3d | 13694 | -0.023(-0.087, 0.04) | 72 | 0.068(0.016, 0.12) | 63 | 0.199(0.073, 0.324) | 94 |
|  | Model 3e | 14128 | -0.023(-0.084, 0.038) | 71 | 0.07(0.017, 0.123) | 66 | 0.197(0.071, 0.324) | 94 |
|  | Model 3f | 14432 | -0.022(-0.085, 0.041) | 73 | 0.069(0.014, 0.123) | 68 | 0.197(0.074, 0.321) | 94 |
|  | Model 3g | 14460 | -0.045(-0.108, 0.017) | 72 | 0.084(0.027, 0.14) | 70 | 0.22(0.081, 0.358) | 95 |
|  | Model 3h | 13741 | -0.02(-0.079, 0.039) | 68 | 0.068(0.015, 0.122) | 66 | 0.196(0.075, 0.316) | 93 |
|  | Model 3i | 14012 | -0.021(-0.085, 0.042) | 73 | 0.071(0.018, 0.125) | 65 | 0.198(0.077, 0.32) | 94 |
|  | Model 3j | 13586 | -0.021(-0.081, 0.038) | 68 | 0.064(0, 0.128) | 76 | 0.203(0.075, 0.331) | 94 |
| TC/ HDL-C^§^ | Model 3 | 14629 | -0.033(-0.063, -0.003) | 11 | 0.19(0.099, 0.282) | 92 | 0.06(0.012, 0.108) | 66 |
|  | Model 3a | 12560 | -0.008(-0.038, 0.021) | 0 | 0.173(0.093, 0.253) | 88 | 0.073(0.02, 0.125) | 67 |
|  | Model 3b | 12110 | -0.018(-0.049, 0.012) | 7 | 0.185(0.091, 0.279) | 91 | 0.046(0.006, 0.087) | 49 |
|  | Model 3c | 14629 | -0.029(-0.061, 0.002) | 14 | 0.189(0.096, 0.283) | 92 | 0.063(0.015, 0.111) | 66 |
|  | Model 3d | 13855 | -0.033(-0.067, 0.001) | 25 | 0.186(0.093, 0.278) | 91 | 0.061(0.014, 0.109) | 65 |
|  | Model 3e | 14279 | -0.03(-0.059, -0.002) | 0 | 0.19(0.096, 0.283) | 92 | 0.062(0.012, 0.113) | 69 |
|  | Model 3f | 14599 | -0.032(-0.062, -0.002) | 12 | 0.189(0.098, 0.28) | 91 | 0.06(0.013, 0.107) | 65 |
|  | Model 3g | 14629 | -0.014(-0.045, 0.017) | 13 | 0.199(0.102, 0.295) | 92 | 0.09(0.024, 0.155) | 82 |
|  | Model 3h | 13883 | -0.034(-0.064, -0.003) | 8 | 0.192(0.101, 0.283) | 91 | 0.061(0.01, 0.112) | 69 |
|  | Model 3i | 14171 | -0.033(-0.064, -0.001) | 16 | 0.19(0.1, 0.279) | 91 | 0.062(0.014, 0.11) | 66 |
|  | Model 3j | 13751 | -0.026(-0.055, 0.004) | 5 | 0.175(0.077, 0.273) | 92 | 0.062(0.015, 0.108) | 63 |
| TG (mmol/l) ^§^ | Model 3 | 14628 | -0.156(-0.209, -0.103) | 70 | 0.19(0.084, 0.296) | 94 | -0.2(-0.334, -0.066) | 96 |
|  | Model 3a | 12558 | -0.138(-0.184, -0.092) | 57 | 0.173(0.084, 0.262) | 90 | -0.187(-0.297, -0.076) | 93 |
|  | Model 3b | 12109 | -0.142(-0.198, -0.086) | 69 | 0.186(0.069, 0.303) | 94 | -0.202(-0.356, -0.048) | 96 |
|  | Model 3c | 14628 | -0.157(-0.21, -0.103) | 70 | 0.19(0.081, 0.3) | 94 | -0.197(-0.33, -0.065) | 96 |
|  | Model 3d | 13854 | -0.155(-0.208, -0.101) | 69 | 0.186(0.074, 0.297) | 94 | -0.196(-0.334, -0.058) | 96 |
|  | Model 3e | 14279 | -0.152(-0.204, -0.1) | 69 | 0.188(0.082, 0.295) | 93 | -0.196(-0.328, -0.064) | 96 |
|  | Model 3f | 14598 | -0.155(-0.207, -0.103) | 69 | 0.19(0.084, 0.296) | 94 | -0.199(-0.333, -0.066) | 96 |
|  | Model 3g | 14628 | -0.101(-0.147, -0.055) | 60 | 0.159(0.074, 0.245) | 90 | -0.167(-0.283, -0.051) | 95 |
|  | Model 3h | 13882 | -0.152(-0.202, -0.102) | 65 | 0.193(0.089, 0.298) | 93 | -0.191(-0.313, -0.068) | 95 |
|  | Model 3i | 14171 | -0.157(-0.21, -0.104) | 69 | 0.189(0.082, 0.296) | 93 | -0.197(-0.331, -0.063) | 96 |
|  | Model 3j | 13750 | -0.143(-0.198, -0.087) | 72 | 0.172(0.062, 0.283) | 94 | -0.197(-0.334, -0.06) | 96 |
| ApoA1 (µmol/l) | Model 3 | 14632 | -0.125(-0.169, -0.081) | 53 | -0.106(-0.167, -0.046) | 77 | -0.126(-0.219, -0.034) | 91 |
|  | Model 3a | 12563 | -0.125(-0.173, -0.076) | 55 | -0.10(-0.158, -0.041) | 72 | -0.127(-0.215, -0.039) | 87 |
|  | Model 3b | 12112 | -0.131(-0.181, -0.08) | 57 | -0.107(-0.178, -0.037) | 80 | -0.126(-0.232, -0.02) | 92 |
|  | Model 3c | 14632 | -0.128(-0.168, -0.088) | 43 | -0.103(-0.164, -0.043) | 78 | -0.127(-0.219, -0.035) | 90 |
|  | Model 3d | 13859 | -0.124(-0.166, -0.082) | 47 | -0.104(-0.161, -0.046) | 74 | -0.125(-0.215, -0.035) | 90 |
|  | Model 3e | 14282 | -0.125(-0.169, -0.081) | 52 | -0.107(-0.169, -0.044) | 79 | -0.126(-0.218, -0.034) | 90 |
|  | Model 3f | 14602 | -0.125(-0.168, -0.082) | 51 | -0.106(-0.165, -0.046) | 77 | -0.125(-0.216, -0.035) | 90 |
|  | Model 3g | 14632 | -0.126(-0.177, -0.076) | 63 | -0.142(-0.226, -0.058) | 88 | -0.123(-0.214, -0.031) | 90 |
|  | Model 3h | 13886 | -0.114(-0.158, -0.07) | 51 | -0.107(-0.163, -0.05) | 73 | -0.117(-0.205, -0.029) | 89 |
|  | Model 3i | 14178 | -0.123(-0.171, -0.075) | 60 | -0.105(-0.162, -0.048) | 74 | -0.125(-0.218, -0.033) | 91 |
|  | Model 3j | 13754 | -0.128(-0.173, -0.083) | 53 | -0.103(-0.166, -0.041) | 78 | -0.127(-0.223, -0.031) | 91 |
| ApoB (µmol/l) | Model 3 | 14621 | -0.079(-0.128, -0.029) | 60 | 0.123(0.058, 0.188) | 79 | 0.123(0.038, 0.208) | 88 |
|  | Model 3a | 12553 | -0.05(-0.083, -0.016) | 11 | 0.104(0.055, 0.153) | 62 | 0.137(0.05, 0.224) | 87 |
|  | Model 3b | 12101 | -0.059(-0.104, -0.014) | 48 | 0.113(0.054, 0.171) | 73 | 0.107(0.028, 0.185) | 85 |
|  | Model 3c | 14621 | -0.074(-0.126, -0.023) | 62 | 0.124(0.058, 0.191) | 80 | 0.126(0.042, 0.211) | 87 |
|  | Model 3d | 13849 | -0.078(-0.131, -0.024) | 63 | 0.12(0.056, 0.184) | 77 | 0.126(0.039, 0.214) | 88 |
|  | Model 3e | 14271 | -0.078(-0.126, -0.03) | 57 | 0.123(0.058, 0.187) | 79 | 0.125(0.038, 0.213) | 88 |
|  | Model 3f | 14591 | -0.078(-0.128, -0.028) | 61 | 0.121(0.056, 0.186) | 79 | 0.123(0.038, 0.207) | 87 |
|  | Model 3g | 14621 | -0.085(-0.133, -0.037) | 57 | 0.126(0.06, 0.192) | 80 | 0.156(0.05, 0.262) | 92 |
|  | Model 3h | 13875 | -0.073(-0.117, -0.03) | 46 | 0.124(0.058, 0.189) | 79 | 0.125(0.038, 0.212) | 88 |
|  | Model 3i | 14169 | -0.077(-0.129, -0.025) | 63 | 0.122(0.059, 0.185) | 77 | 0.124(0.042, 0.206) | 87 |
|  | Model 3j | 13743 | -0.073(-0.121, -0.025) | 55 | 0.114(0.044, 0.184) | 81 | 0.128(0.041, 0.214) | 88 |
| ApoB/A1 | Model 3 | 14621 | 0.002(-0.027, 0.032) | 0 | 0.158(0.081, 0.235) | 87 | 0.154(0.047, 0.261) | 93 |
|  | Model 3a | 12553 | 0.028(-0.006, 0.063) | 19 | 0.138(0.069, 0.207) | 82 | 0.163(0.058, 0.268) | 92 |
|  | Model 3b | 12101 | 0.022(-0.011, 0.054) | 10 | 0.149(0.07, 0.228) | 86 | 0.14(0.031, 0.248) | 93 |
|  | Model 3c | 14621 | 0.007(-0.026, 0.041) | 18 | 0.157(0.079, 0.236) | 87 | 0.157(0.05, 0.264) | 93 |
|  | Model 3d | 13849 | 0.002(-0.032, 0.037) | 21 | 0.155(0.08, 0.231) | 85 | 0.157(0.05, 0.264) | 93 |
|  | Model 3e | 14271 | 0.003(-0.027, 0.033) | 0 | 0.158(0.079, 0.238) | 87 | 0.156(0.048, 0.264) | 93 |
|  | Model 3f | 14591 | 0.003(-0.028, 0.034) | 7 | 0.156(0.08, 0.233) | 86 | 0.153(0.048, 0.259) | 93 |
|  | Model 3g | 14621 | 0.004(-0.031, 0.039) | 25 | 0.18(0.084, 0.276) | 91 | 0.179(0.054, 0.303) | 95 |
|  | Model 3h | 13875 | 0.002(-0.028, 0.032) | 0 | 0.16(0.082, 0.237) | 86 | 0.152(0.044, 0.259) | 93 |
|  | Model 3i | 14169 | 0.003(-0.03, 0.037) | 18 | 0.156(0.082, 0.23) | 85 | 0.155(0.051, 0.259) | 92 |
|  | Model 3j | 13743 | 0.007(-0.022, 0.037) | 0 | 0.148(0.068, 0.229) | 87 | 0.157(0.048, 0.265) | 93 |
| ALT (µkat/l) ^§^ | Model 3 | 14600 | -0.079(-0.121, -0.037) | 48 | 0.099(0.041, 0.157) | 76 | -0.036(-0.088, 0.017) | 69 |
|  | Model 3a | 12537 | -0.071(-0.11, -0.033) | 29 | 0.094(0.04, 0.147) | 67 | -0.032(-0.097, 0.032) | 75 |
|  | Model 3b | 12080 | -0.077(-0.128, -0.026) | 59 | 0.095(0.03, 0.16) | 77 | -0.036(-0.096, 0.025) | 74 |
|  | Model 3c | 14600 | -0.085(-0.132, -0.038) | 57 | 0.1(0.04, 0.159) | 77 | -0.033(-0.085, 0.018) | 68 |
|  | Model 3d | 13827 | -0.082(-0.123, -0.041) | 41 | 0.103(0.047, 0.159) | 72 | -0.032(-0.083, 0.019) | 65 |
|  | Model 3e | 14250 | -0.076(-0.113, -0.039) | 31 | 0.096(0.041, 0.151) | 73 | -0.033(-0.085, 0.019) | 68 |
|  | Model 3f | 14570 | -0.078(-0.12, -0.037) | 46 | 0.1(0.042, 0.157) | 75 | -0.036(-0.087, 0.016) | 68 |
|  | Model 3g | 14600 | -0.059(-0.09, -0.029) | 0 | 0.087(0.036, 0.138) | 68 | -0.013(-0.052, 0.025) | 40 |
|  | Model 3h | 13857 | -0.066(-0.11, -0.023) | 48 | 0.095(0.041, 0.149) | 71 | -0.025(-0.072, 0.023) | 61 |
|  | Model 3i | 14151 | -0.077(-0.117, -0.038) | 40 | 0.098(0.042, 0.154) | 73 | -0.035(-0.086, 0.016) | 67 |
|  | Model 3j | 13722 | -0.074(-0.112, -0.036) | 33 | 0.086(0.03, 0.142) | 72 | -0.032(-0.085, 0.021) | 68 |
| AST (µkat/l) ^§^ | Model 3 | 14409 | -0.125(-0.192, -0.059) | 77 | 0.05(0.008, 0.093) | 48 | -0.068(-0.148, 0.013) | 86 |
|  | Model 3a | 12386 | -0.123(-0.184, -0.061) | 69 | 0.043(0.007, 0.079) | 19 | -0.065(-0.155, 0.025) | 86 |
|  | Model 3b | 11896 | -0.12(-0.196, -0.044) | 79 | 0.051(0.002, 0.1) | 54 | -0.067(-0.155, 0.021) | 86 |
|  | Model 3c | 14409 | -0.134(-0.208, -0.06) | 81 | 0.05(0.007, 0.093) | 49 | -0.066(-0.147, 0.015) | 86 |
|  | Model 3d | 13644 | -0.126(-0.188, -0.064) | 71 | 0.051(0.011, 0.09) | 36 | -0.061(-0.139, 0.016) | 84 |
|  | Model 3e | 14064 | -0.122(-0.183, -0.061) | 72 | 0.047(0.006, 0.089) | 46 | -0.067(-0.145, 0.012) | 85 |
|  | Model 3f | 14379 | -0.124(-0.189, -0.06) | 75 | 0.051(0.009, 0.092) | 45 | -0.066(-0.144, 0.012) | 85 |
|  | Model 3g | 14409 | -0.111(-0.164, -0.057) | 62 | 0.028(-0.002, 0.059) | 0 | -0.042(-0.102, 0.019) | 73 |
|  | Model 3h | 13677 | -0.107(-0.164, -0.05) | 67 | 0.044(0.003, 0.084) | 43 | -0.049(-0.116, 0.017) | 78 |
|  | Model 3i | 13967 | -0.121(-0.184, -0.059) | 73 | 0.048(0.007, 0.088) | 41 | -0.066(-0.147, 0.015) | 86 |
|  | Model 3j | 13530 | -0.121(-0.187, -0.054) | 76 | 0.044(-0.001, 0.09) | 53 | -0.068(-0.151, 0.014) | 86 |
| GGT (µkat/l) ^§^ | Model 3 | 14620 | -0.193(-0.301, -0.085) | 93 | 0.124(0.031, 0.218) | 92 | -0.111(-0.213, -0.009) | 93 |
|  | Model 3a | 12551 | -0.187(-0.293, -0.081) | 92 | 0.122(0.032, 0.211) | 89 | -0.106(-0.207, -0.005) | 91 |
|  | Model 3b | 12105 | -0.184(-0.304, -0.063) | 94 | 0.119(0.012, 0.227) | 93 | -0.112(-0.225, 0) | 94 |
|  | Model 3c | 14620 | -0.194(-0.302, -0.085) | 93 | 0.125(0.031, 0.218) | 92 | -0.11(-0.212, -0.008) | 93 |
|  | Model 3d | 13847 | -0.192(-0.291, -0.093) | 92 | 0.124(0.033, 0.215) | 91 | -0.101(-0.196, -0.006) | 92 |
|  | Model 3e | 14271 | -0.192(-0.298, -0.087) | 93 | 0.121(0.03, 0.212) | 91 | -0.108(-0.207, -0.009) | 93 |
|  | Model 3f | 14590 | -0.19(-0.294, -0.086) | 93 | 0.123(0.03, 0.216) | 91 | -0.108(-0.205, -0.01) | 92 |
|  | Model 3g | 14620 | -0.165(-0.254, -0.077) | 89 | 0.091(0.021, 0.16) | 85 | -0.063(-0.129, 0.004) | 83 |
|  | Model 3h | 13874 | -0.173(-0.266, -0.079) | 91 | 0.119(0.035, 0.203) | 90 | -0.091(-0.175, -0.007) | 90 |
|  | Model 3i | 14163 | -0.191(-0.298, -0.084) | 93 | 0.117(0.027, 0.207) | 91 | -0.108(-0.21, -0.006) | 93 |
|  | Model 3j | 13746 | -0.181(-0.289, -0.073) | 93 | 0.11(0.014, 0.206) | 92 | -0.107(-0.21, -0.004) | 93 |
| HbA1c (mmol/mol) | Model 3 | 15019 | -0.015(-0.045, 0.014) | 3 | 0.036(-0.006, 0.077) | 54 | 0.032(-0.009, 0.072) | 50 |
|  | Model 3a | 12903 | -0.015(-0.049, 0.019) | 13 | 0.031(-0.005, 0.067) | 31 | 0.025(-0.013, 0.062) | 29 |
|  | Model 3b | 12266 | -0.013(-0.045, 0.02) | 5 | 0.04(-0.003, 0.083) | 48 | 0.034(-0.011, 0.08) | 53 |
|  | Model 3c | 15019 | -0.02(-0.05, 0.009) | 0 | 0.035(-0.005, 0.076) | 52 | 0.031(-0.01, 0.072) | 51 |
|  | Model 3d | 14206 | -0.018(-0.054, 0.018) | 29 | 0.035(-0.003, 0.074) | 45 | 0.03(-0.009, 0.07) | 46 |
|  | Model 3e | 14808 | 0(-0.033, 0.034) | 52 | 0.022(-0.01, 0.055) | 53 | 0.052(0.009, 0.095) | 73 |
|  | Model 3f | 14989 | -0.015(-0.045, 0.015) | 6 | 0.036(-0.006, 0.077) | 55 | 0.031(-0.009, 0.071) | 48 |
|  | Model 3g | 15019 | -0.017(-0.049, 0.016) | 16 | 0.036(-0.007, 0.079) | 57 | 0.04(-0.006, 0.087) | 61 |
|  | Model 3h | 14250 | -0.016(-0.045, 0.014) | 0 | 0.037(-0.003, 0.077) | 51 | 0.027(-0.009, 0.063) | 37 |
|  | Model 3i | 14029 | -0.015(-0.049, 0.02) | 26 | 0.033(-0.007, 0.074) | 52 | 0.029(-0.008, 0.065) | 39 |
|  | Model 3j | 14162 | -0.011(-0.041, 0.019) | 0 | 0.03(-0.009, 0.068) | 44 | 0.037(-0.009, 0.082) | 57 |
| CRP (nmol/l) ^§^ | Model 3 | 14623 | -0.1(-0.144, -0.055) | 51 | 0.15(0.096, 0.205) | 71 | 0.004(-0.028, 0.036) | 17 |
|  | Model 3a | 12554 | -0.095(-0.143, -0.046) | 52 | 0.153(0.089, 0.218) | 76 | 0(-0.037, 0.036) | 20 |
|  | Model 3b | 12110 | -0.097(-0.147, -0.047) | 54 | 0.15(0.086, 0.214) | 75 | 0.005(-0.033, 0.042) | 29 |
|  | Model 3c | 14623 | -0.098(-0.139, -0.057) | 41 | 0.15(0.096, 0.204) | 70 | 0.006(-0.028, 0.039) | 24 |
|  | Model 3d | 13851 | -0.099(-0.142, -0.056) | 44 | 0.148(0.101, 0.196) | 59 | 0.008(-0.028, 0.044) | 32 |
|  | Model 3e | 14274 | -0.099(-0.143, -0.054) | 50 | 0.15(0.094, 0.206) | 71 | 0.003(-0.032, 0.037) | 26 |
|  | Model 3f | 14593 | -0.098(-0.138, -0.057) | 41 | 0.149(0.095, 0.203) | 70 | 0.005(-0.026, 0.035) | 12 |
|  | Model 3g | 14623 | -0.081(-0.124, -0.037) | 47 | 0.143(0.092, 0.194) | 65 | 0.032(-0.002, 0.065) | 22 |
|  | Model 3h | 13877 | -0.095(-0.138, -0.052) | 44 | 0.149(0.096, 0.203) | 68 | 0.005(-0.023, 0.034) | 0 |
|  | Model 3i | 14178 | -0.095(-0.136, -0.054) | 53 | 0.124(0.069, 0.178) | 76 | 0(-0.033, 0.034) | 36 |
|  | Model 3j | 13750 | -0.091(-0.134, -0.048) | 46 | 0.138(0.08, 0.196) | 73 | 0.008(-0.027, 0.043) | 27 |

^δ^ Values represent standardised difference (in SD unit of the metabolic marker) per 1 SD of each saturated fatty acid (SFA) group: odd-chain SFA (C15:0 + C17:0), even-chain SFA (C14:0 + C16:0 + C18:0), very-long-chain SFA (C20:0 + C22:0 + C23:0 + C24:0). The confidence intervals were corrected for multiple testing based on a familywise error rate of 5%.

^§^ TG, TC/ HDL-C, ALT, AST, GGT and CRP were log-transformed;

Sensitivity analyses were conducted based on model 3 (Supplemental Table 2-4).

Model 3, adjusted for age, sex, centre, body-mass index, physical activity, smoking status, alcohol drinking, educational level and total energy intake.

Model 3a, model 3 plus a variety of dietary factors, including dietary carbohydrate intake, dairy intake, red and processed meat intake, fruit/vegetable intake, olive oil and vegetable oil intake;

Model 3b, model 3, excluding participants with HbA1c>6.5%;

Model 3c, model 3 plus self-report heart disease, stroke and cancer as covariates;

Model 3d, model 3, excluding participants with self-report heart disease, stroke or cancer;

Model 3e, model 3 excluding participants with self-reported hyperlipidaemia;

Model 3f, model 3 plus self-reported hyperlipidaemia as a covariate;

Model 3g, model 3 plus mutual adjustment for the other two saturated fatty acid groups;

Model 3h, model 3 excluding participants with heavy alcohol drinking (> 95th percentiles in the subcohort [>50.2 g/d])

Model 3i, model 3 excluding participants with CRP≥ 95.2 nmol/l (10 mg/l);

Model 3j, model 3, replacing BMI with waist circumference.

All the analyses were conducted in each country and then pooled by random-effects meta-analysis. I^2^ represent heterogeneity across the countries.

TC, total cholesterol; HDL-C, high-density lipoprotein cholesterol; LDL-C, low-density lipoprotein cholesterol; TG, triglycerides; ApoA1, apolipoprotein A-1; ApoB, apolipoprotein B; ALT, alanine transaminase; GGT, gamma glutamyl transferase; AST, aspartate transaminase; HbA1c, haemoglobin A1c; CRP, C-reactive protein.

**Figure S1 Association of plasma phospholipid saturated fatty acid groups with LDL-C, TG and GGT by countries: EPIC-InterAct Study**

**
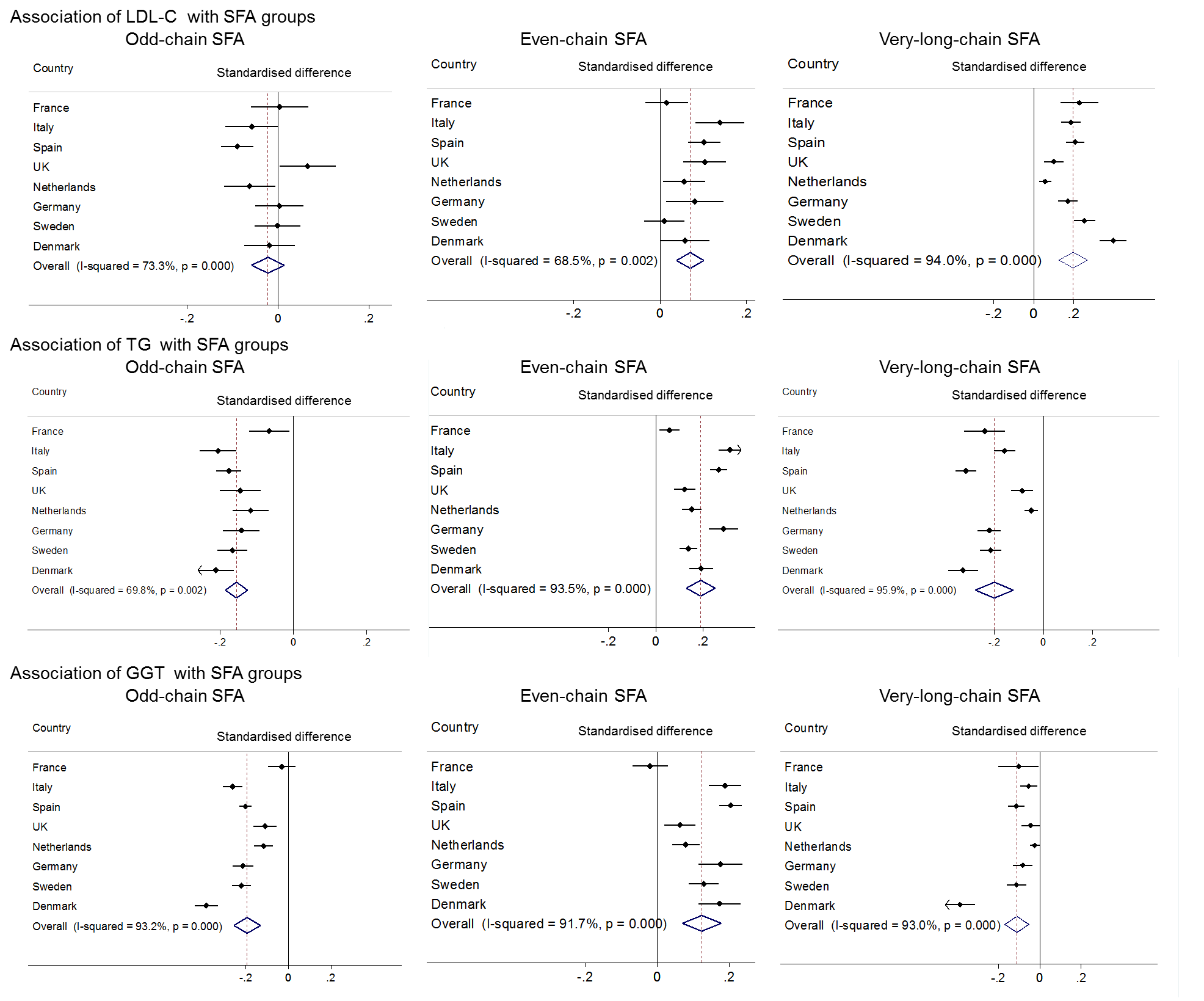
**

**Figure S2 Association of plasma phospholipid saturated fatty acid groups with metabolic markers by subgroups of alcohol intake, BMI and sex: EPIC-InterAct Study**


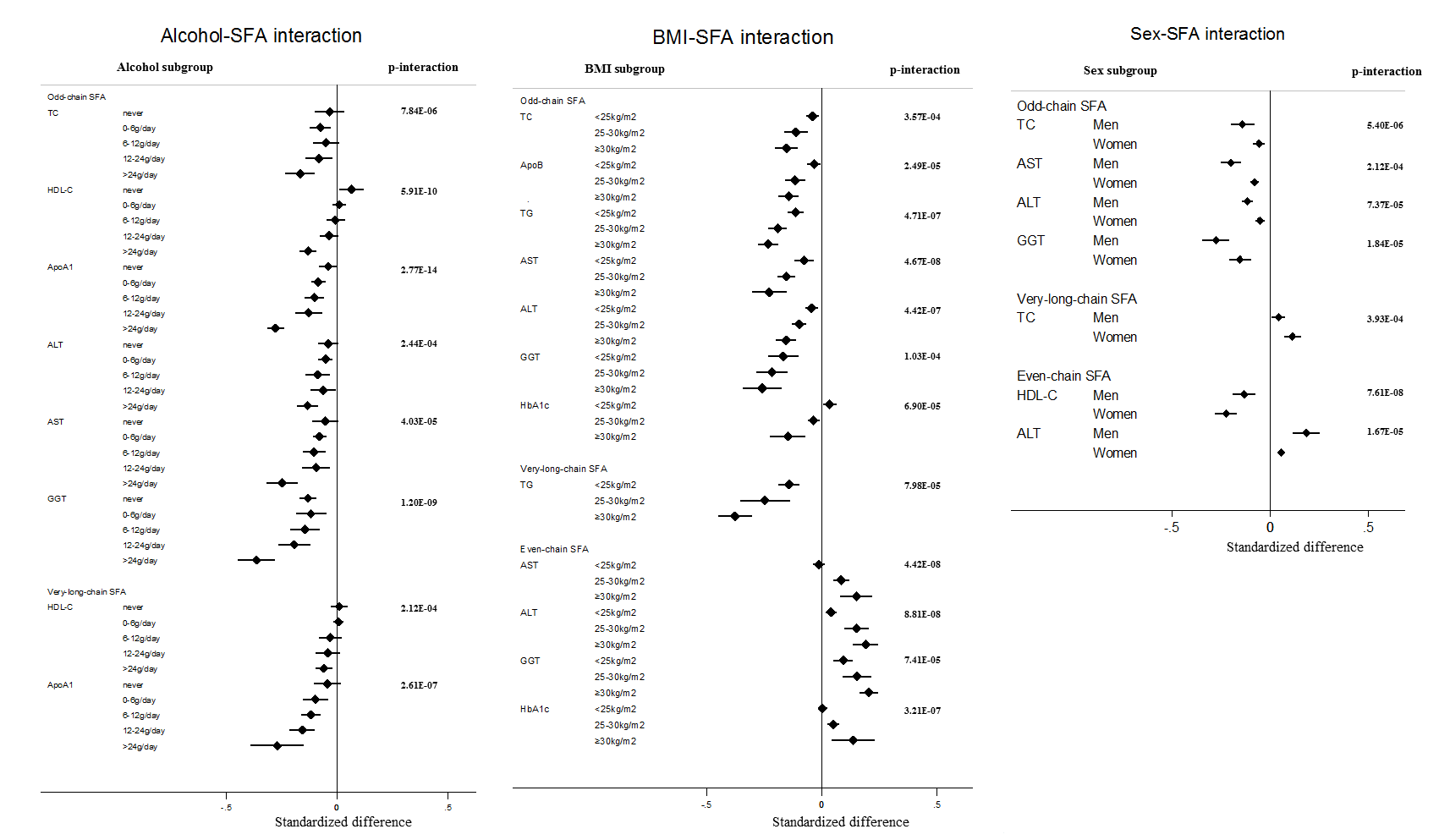

Supplement: Additional file 1: Table S1. — Population characteristics by the eight participating countries: EPIC-InterAct study. Table S2. Distribution of plasma metabolic markers by quartiles of plasma phospholipid saturated fatty acid (SFA) groups: EPIC-InterAct study. Table S3. Pairwise correlation of plasma phospholipid SFAs in the subcohort of EPIC-InterAct study. Table S4. Association of plasma phospholipid odd-chain SFAs (per 1 SD difference) with metabolic markers: EPIC-InterAct study. Table S5. Association of plasma phospholipid even-chain SFAs (per 1 SD difference) with metabolic markers: EPIC-InterAct study. Table S6. Association of plasma phospholipid very-long-chain SFAs (per 1 SD difference) with metabolic markers: EPIC-InterAct study. Table S7. Sensitivity analysis for the association of plasma phospholipid SFA groups and metabolic markers: EPIC-InterAct study. Figure S1. Association of plasma phospholipid SFA groups with LDL-C, TG and GGT by countries: EPIC-InterAct study. Figure S2. Association of plasma phospholipid SFA groups with metabolic markers by subgroups of alcohol intake, BMI and sex: EPIC-InterAct study. (DOCX 321 kb) [file 12916_2017_968_MOESM1_ESM.docx]
